# ﻿The Trichoptera of Panama XXII. Sixteen new microcaddisfly species (Trichoptera, Hydroptilidae)

**DOI:** 10.3897/zookeys.1174.107314

**Published:** 2023-08-08

**Authors:** Steven C. Harris, Brian J. Armitage

**Affiliations:** 1 Department of Biology and Environmental Sciences, Western Pennsylvania University, Clarion, PA 16214, USA Western Pennsylvania University Clarion United States of America; 2 Museo de Peces de Agua Dulce e Invertebrados, Universidad Autónoma de Chiriquí, David, Panama Universidad Autónoma de Chiriquí David Panama

**Keywords:** Biological diversity, Neotropics, protected areas, regional affiliation

## Abstract

Sixteen new species of microcaddisflies (Trichoptera, Hydroptilidae) from Panama are herein described and illustrated. The majority of these were collected during surveys of Panama’s national parks and protected areas during 2017 and 2018, employing both UV and Malaise traps. The new species include: *Alisotrichiaeisbergae***sp. nov.**, *Angrisanoiabokota***sp. nov.**, *Brediniaparaespinosa***sp. nov.**, *Cerasmatrichiagarfioza***sp. nov.**, *Cerasmatrichiaveraguasensis***sp. nov.**, *Costatrichiacalovebora***sp. nov.**, *Metrichiacalovebora***sp. nov.**, *Metrichiacascada***sp. nov.**, *Metrichiachiriquiensis***sp. nov.**, *Metrichiaescobilla***sp. nov.**, *Metrichialeahae***sp. nov.**, *Metrichiatatianae***sp. nov.**, *Ochrotrichiaconejoreja***sp. nov.**, *Ochrotrichiaparaflagellata***sp. nov.**, *Oxyethirapehrssonae***sp. nov.**, and *Zumatrichiaculebra***sp. nov.** In total, 506 Trichoptera species are now recorded for the Republic of Panama, distributed among 15 families and 56 genera.

## ﻿Introduction

Until the last 27 years, the insect order Trichoptera (caddisflies) was poorly known in Panama, both in terms of diversity and distribution (Armitage and Cornejo 2015). In general, repeated collections were made in relatively few locations. Aguila (1992) published the first list of caddisflies (Insecta, Trichoptera) from Panama, including 168 species in 13 families and 39 genera. From that publication and through 2014, six genera and 78 species were added to Panama’s caddisfly fauna by a number of researchers, bringing the total to 246 species distributed among 13 families and 45 genera. Beginning in 2015 and continuing into 2022, two families, 11 genera, and 241 new species and new country records of caddisflies have been added to Panama’s fauna (Armitage et al. 2015, 2021 for a summary; Armitage et al. 2022a, 2022b; Thomson et al. 2022; Harris et al. 2023 for recent additions). In this paper we describe and illustrate an additional 16 new species of microcaddisflies. Now the total of known caddisflies from the Republic of Panama is 506 species distributed among 15 families and 56 genera.

Starting in 2017, field sampling for a new biodiversity initiative in the Republic of Panama was begun under the management of Panama’s Ministerio de Ambiente (MiAmbiente). The focus was on biodiversity of the country’s national parks and protected areas. Designated “Proyecto Sistema de Producción Sostenible Conservación de la Biodiversidad (PSPSCB; http://produccionsostenibleybiodiversidad.org/proyecto/)”, this initiative was funded by the World Bank. M’Ambiente collaborated with the Instituto Conmemorativo Gorgas de Estudios de la Salud (Gorgas Institute), and their Colección Zoológica Dr. Eustorgio Méndez (COZEM) to execute the work. These biodiversity surveys are included under the framework of the “Sistema Nacional de Información y Monitoreo de la Diversidad Biológica”, or National Biological Diversity Information and Monitoring System, to better understand the country’s biodiversity. One of the components of this new project involved surveys for aquatic invertebrates. The majority of the new species described in this paper was collected in the framework of this project.

During 2017, samples were taken in four national parks: Omar Torrijos Herrera (**PNGDOTH**), Santa Fe (**PNSF**), Volcán Barú (**PNVB**), and La Amistad International (**PILA**). In 2018, Altos de Campana National Park (**PNAC**) was surveyed. Finally, in 2019, collections were made in Bosque Protector Palo Seco (**BPPS**). In this paper, we describe species from PNVB, PNSF, and PNAC. Previously, new species and general results from Omar Torrijos Herrera General Division National Park were published in Armitage and Harris (2020) and Armitage et al. (2021), respectively. New species from BPPS are described in a separate paper (Harris et al. 2023). No taxa new to science resulted from samples taken in PILA.

The Aquatic Invertebrate Research Group (AIRG) at the Universidad Autónoma de Chiriquí (UNACHI) and its Museo de Peces de Agua Dulce e Invertebrados (MUPADI) is currently focused on increasing our knowledge of Trichoptera (caddisflies) and Plecoptera (stoneflies) in Panama. Toward that goal, it has secured registered projects for these two orders of aquatic insects. Most of the new species described in this paper were collected before the establishment of AIRG. However, current AIRG and MUPADI personnel were involved with the PSPSCB project throughout, and continue to be involved in the publication and documentation of results from that effort.

## ﻿Materials and methods

Both Malaise and UV light traps were used for collecting aquatic insects from streams in the national parks and protected areas of Panama. Single, overnight collections were made using UV light traps (Calor and Mariano 2012). Multiple-night collections were made employing Malaise traps over four-day periods. Specimens were prepared and examined following standard methods outlined in Blahnik and Holzenthal (2004). Male genitalia were soaked in 5% KOH overnight, and washed in weakly acidified alcohol prior to examination under a dissecting scope.

Morphological terminology used for male genitalia generally follows that of Marshall (1979) and classification within the Hydroptilidae follows Thomson (2023). Paired structures are discussed in the singular for simplicity. Although technically segments V through X are not part of the genitalia, traditionally descriptions of segments VII–X have been included under the genitalia heading. We follow that practice here. If segments V and VI have distinct features, they are discussed under the male description. Total length of specimens provided in descriptions represents the length from the tip of the head to the tip of the forewing. Altitude values are given in meters above sea level (m a.s.l.). Maps were created in QGIS software, version 3.28.5-Firenze.

Holotypes listed in this publication are deposited in the Universidad de Panamá Museo de Invertebrados (**MIUP**) or MUPADI. Paratypes and other specimens are deposited in MUPADI, the University of Minnesota’s Neotropical Insect Collection (**UMSP**), or the second author’s reference collection (**SCH**). The genera and species listed below are in alphabetic order.

### ﻿Field sites (National Parks and Protected Areas)

**Figure 1.**
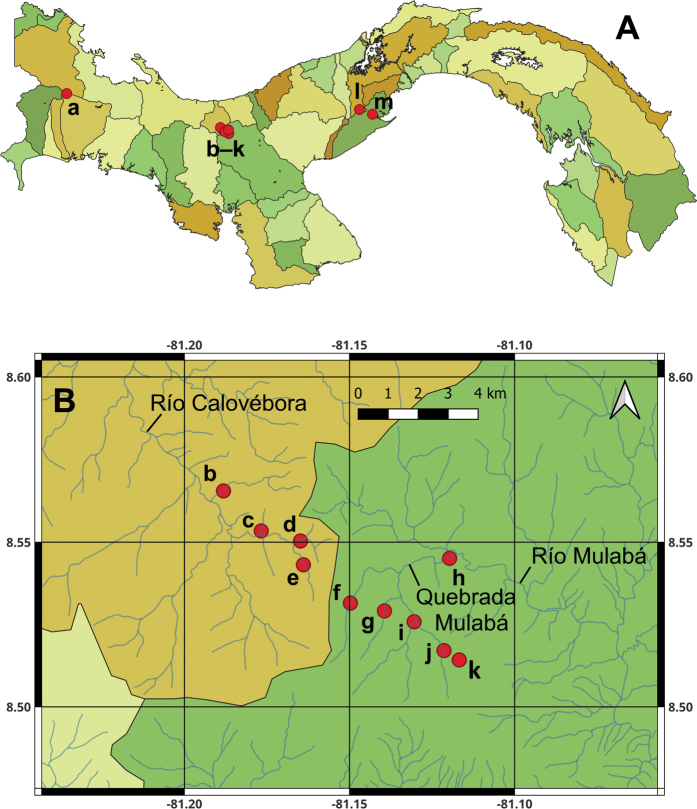
Maps: **A** map of Panama with an overlay of the 52 major watersheds (cuencas) **B** map of central Panama showing collection locations in the Rio Calovebora and Rio Mulabá drainages. Abbreviations: a– Quebrada del Guayabo; b– Río Piedra de Moler; c– Quebrada sin nombre; d– Quebrada sin nombre; e– Río Calovébora; f– Quebrada Tercer Brazo Mulabá; g– Quebrada Segundo Brazo Mulabá; h– Quebrada Mulabá; i– Quebrada Primer Brazo Mulabá; j– Quebrada Primer Brazo Mulabá; k– Lago cabaña Alto de Piedra; l– Río Cacaito; m– Río Sajalices.

Field sites were located in the following national parks and protected areas in Panama (Fig. 1).

**Parque Nacional Volcán Barú (Volcán Barú NP or PNVB)**—Attached to the southeast side of PILA, Volcán Barú National Park covers 14,300 ha west and northwest of Boquete, Panama. It includes Volcán Barú, the highest volcano in Panama (3,478 m a.s.l.). The vegetation ranges from montane rain forests at the volcano’s base to humid montane forests toward its peak.

**Parque Nacional Santa Fe (Santa Fe NP or PNSF)**—Located in the upper portion of the Santa Maria River basin in Veraguas Province, Santa Fe National Park lies near the Continental Divide and encompasses 72,636 ha. Occupying land on both the Caribbean and Pacific slopes, more than 95% of the park’s area is covered with tree species which are evergreen, maintaining their leaves all year round. The Río Calovébora and its tributaries drain into the Caribbean Sea, whereas the Río Mulabá and its tributaries lie on the Pacific slope.

**Parque Nacional Altos de Campana (Altos Campana NP or PNAC)**—Established in 1966, Altos de Campana National Park is the oldest park in Panama. Covering 1,950 ha, the park lies on the Pacific slope of Panama and is covered, in part, by humid tropical and premontane forests.

All of the national parks selected here are protected from logging and agriculture. The streams sampled under this project are 1^st^ to 3^rd^ order in size, are of good water quality, and are bordered by extensive, forested riparian corridors. Almost all of the streams are found in major watersheds (cuencas), including Cuencas 105, 115, 132, and 138, which are characterized in Cornejo et al. (2017). Cuenca 097 (Río Calovébora watershed; Caribbean drainage for Santa Fe National Park), however, is not included in that book.

## ﻿Results

Collections from selected national parks and protected areas were made during the years 2017–2019 and yielded 16 new species of microcaddisflies.

### ﻿Taxonomy

#### Genus *Alisotrichia* Flint

The genus *Alisotrichia* (Leucotrichinae, Alisotrichiini) is represented by 61 extant species. Restricted to the New World, its distribution ranges from the southwestern United States south to Venezuela; the genus also occurs in the Caribbean Sea’s Antilles chain of islands (Holzenthal and Calor 2017). The eight species known from Panama include recently described species (Harris and Armitage 2019; Armitage and Harris 2020). Herein we add a ninth species for Panama.

##### ﻿ Alisotrichia eisbergae sp. nov.

Taxon classificationAnimaliaTrichopteraHydroptilidae

495EB450-8B81-5496-B0C4-918B36F0C29C

https://zoobank.org/BB6252E9-C6EC-4863-9596-7E1DA073C64F

Fig. 2

###### Type locality.

**Panama: Veraguas Province**: Cuenca 097; Santa Fe District; Santa Fe NP; Río Piedra de Moler; PSPSCB-PNSF-C097-2017-012; 8.56553°N, 81.18817°W; 340 m a.s.l.

###### Type material.

***Holotype*: male, Panama: Veraguas Province**: Cuenca 097; Santa Fe District; Santa Fe NP; Río Piedra de Moler; PSPSCB-PNSF-C097-2017-012; 8.56553°N, 81.18817°W; 340 m a.s.l.; UV light trap; A. Cornejo, T. Ríos, E. Álvarez, C. Nieto, leg.; 20.iv.2017; MIUP-001-T-2023 (in alcohol). ***Paratypes***: same data as for holotype; 4 males; MIUP (in alcohol).

###### Other material examined.

**Panama: Veraguas Province** • 1 male; Cuenca 132; Santa Fe District; Santa Fe NP; Quebrada Primer Brazo Mulabá; PSPSCB-PNSF-C132-2017-007; 8.52577°N, 81.13045°W; 623 m a.s.l.; Malaise trap; A. Cornejo, T. Ríos, E. Álvarez, C. Nieto, leg.; 19–23.iv.2017; MIUP • ibid., 23 males; Río Calovébora; PSPSCB-NPSF-C-097-2017-005; 8.54318°N, 81.16398°W; 536 m a.s.l.; Malaise trap; T. Ríos, E. Álvarez, C. Nieto, leg.; 19–23.iv.2017; MUPADI • ibid., 2 males; Quebrada sin nombre; PSPSCB-NPSF-C-097-2017-006; 8.55038°N, 81.16486°W; 515 m a.s.l.; Malaise trap; A. Cornejo,T. Ríos, E. Álvarez, C. Nieto, leg.; 23–27.iv.2017; MIUP • ibid., 1 male; Quebrada sin nombre; PSPSCB-NPSF-C-097-2017-011; 8.55343°N, 81.17675°W; 395 m a.s.l.; UV light trap; A. Cornejo, T. Ríos, E. Álvarez, C. Nieto, leg.; 20.iv.2017; MIUP. **Panama Oeste Province** • 1 male; Cuenca 115; Capira District; Alto Campana NP; Río Cacaito; PSPSCB-PNAC-C115-2018-028; 8.71650°N, 80.00740°W; 497 m a.s.l.; Malaise trap; T. Ríos, Y. Aguirre, E. Pérez; 23–31.v.2018; MUPADI.

###### Diagnosis.

This species is placed in the *Alisotrichiaorophila* group of Oláh and Flint (2012) on the basis of the dorsolateral process from segment VIII which bears an elongate seta, with closest similarity to *A.neblina* Harris & Flint and *A.coclensis* Armitage & Harris. Like these species, *A.eisbergae* sp. nov. has posterior processes and spines from the margin of segment VIII, but the new species is distinguished by the shape of these spines and by the phallic structure.

**Figure 2.**
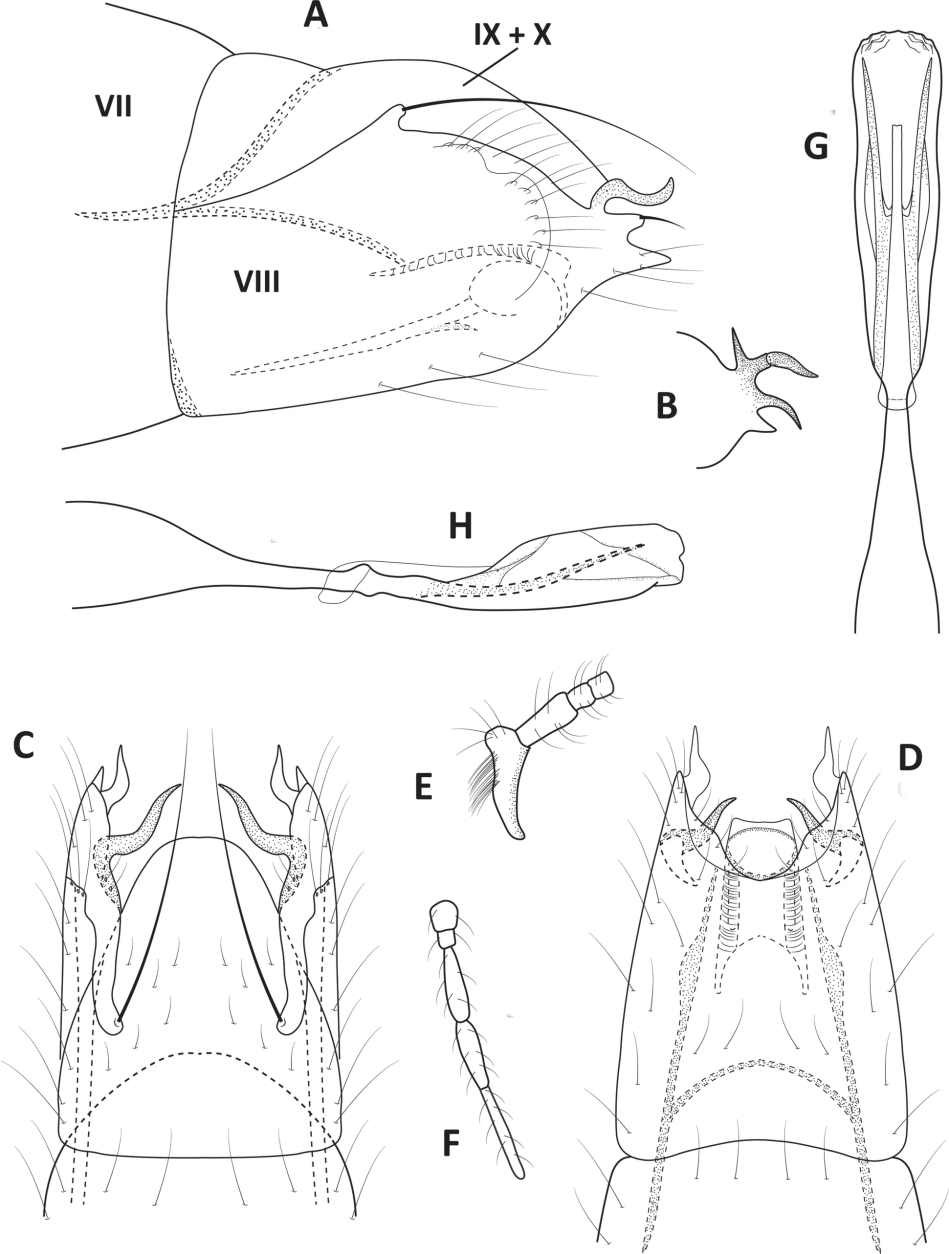
*Alisotrichiaeisbergae* sp. nov., male holotype, genitalia **A** left lateral **B** variation in apex of segment VIIl **C** dorsal **D** ventral **E** antenna, basal segments **F** maxillary palp **G** phallus, dorsal **H** phallus, left lateral.

###### Description.

**Male.** Total length 1.6–1.8 mm, 17 antennal segments, scape enlarged, pedicel twice as long as proximal flagellomeres, maxillary palp with five segments, brown in alcohol with no obvious patterns on wings. **G*enitalia*.** Abdominal segment VII annular without ventromesal process. Segment VIII incomplete dorsally, setal-bearing process dorsally, pair of elongate processes posteriorly; in dorsal view, elongate seta emanating from apex of elongate, narrow, lateral lobes, posterior spines from mesal margins; in ventral view emarginated posteriorly with mesal spines. Segment IX elongate, anteriorly narrowing to short apodeme; in dorsal view narrowing to rounded distal margin; in ventral view narrow, mesal narrow process with crenulate outer margins. Segment X apparently fused with IX and indistinct laterally. Phallus tubular, narrowing at midlength, posteriorly with pair of lateral thin, lateral bands adjacent to and extending beyond the ejaculatory duct; in lateral view wide basally and distally, narrow, spinelike band mesally.

###### Distribution.

Panama.

###### Etymology.

This species is named for Ms. Deborah Eisberg of Boquete, Panama in recognition and thanks for her support of our research program. The species name is a female noun in the genitive case.

###### Remarks.

The pair of elongate processes on segment VIII differed slightly in the specimen from the Río Cacaito (Panama Oeste Province) compared to the other specimens examined.

#### Genus *Angrisanoia* Ozdikmen

*Angrisanoia* is a very small genus in the Ochrotrichiinae. The five species currently assigned to this genus are distributed from Venezuela and French Guiana south to Argentina. The presence and range extension of *Angrisanoia* as a new record for Panama was previously published (Armitage et al. 2020). Herein we describe and figure the new species supporting that new genus record.

##### ﻿ Angrisanoia bokota sp. nov.

Taxon classificationAnimaliaTrichopteraHydroptilidae

4C1530B9-BA55-5A22-BC65-888430F9B30E

https://zoobank.org/F57D74C7-B5F5-409A-89C7-510486AE5C66

Figs 3 , 4

###### Type Locality.

**Panama: Veraguas Province**: Cuenca 132; Santa Fe District; Santa Fe NP; Quebrada Primer Brazo Mulabá; Isleta; PSPSCB-PNSF-C132-2017-015; 8.54513°N, 81.11970°W; 412 m a.s.l.

###### Type material.

***Holotype*: male, Panama: Veraguas Province**: Cuenca 132; Santa Fe District; Santa Fe NP; Quebrada Primer Brazo Mulabá; Isleta; PSPSCB-PNSF-C132-2017-015; 8.54513°N, 81.11970°W; 412 m a.s.l.; UV light trap; T. Ríos, E. Álvarez, C. Nieto, leg.; 22.iv.2017; MIUP-002-T-2023 (in alcohol).

###### Other material examined.

**Panama: Veraguas Province** • 1 male, Cuenca 132; Santa Fe District; Santa Fe NP; Quebrada Primer Brazo Mulabá; PSPSCB-PNSF-C132-2017-007; 8.52577°N, 81.13045°W; 623 m a.s.l.; UV light trap; A. Cornejo, T. Ríos, E. Álvarez, C. Nieto, leg.; 20.iv.2017; MIUP • ibid., 1 male, Río Piedra de Moler; PSPSCB-PNSF-C097-2017-012; 8.56553°N, 81.18817°W; 340 m a.s.l.; UV light trap; A. Cornejo, T. Ríos, E. Álvarez, C. Nieto, leg.; 20.iv.2017; MUPADI.

###### Diagnosis.

The new species is most similar to *A.acuti* (Angrisano & Spanga) and *A.cebollati* (Angrisano) in having segment IX with an elongate posterior extension, which is deeply divided distally. It differs from these species in having the inferior appendage narrowing distally in lateral view, and curving ventrad, with clusters of heavy spines from the inner margin.

**Figure 3.**
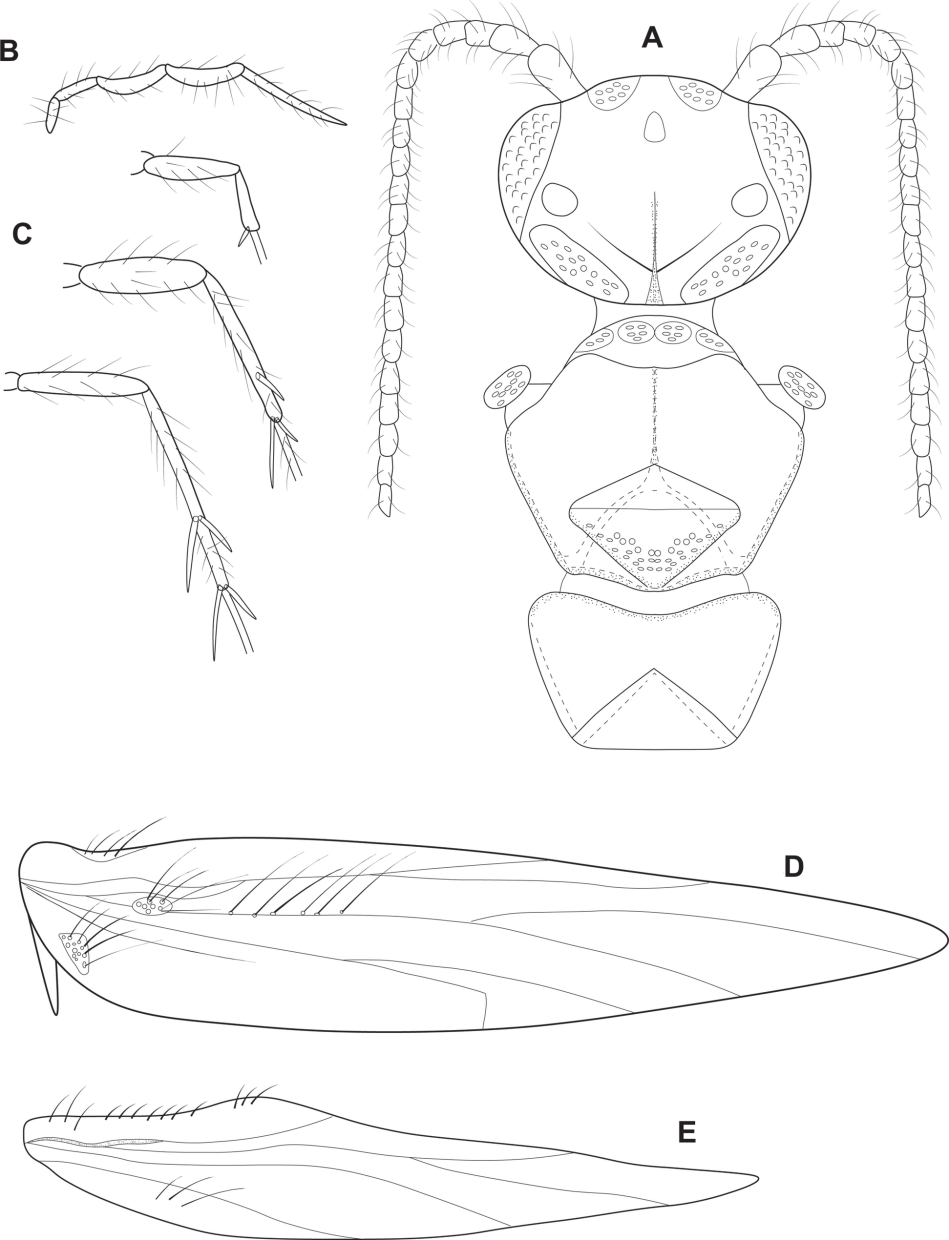
*Angrisanoiabokota* sp. nov., male holotype **A** head and thorax, dorsal **B** maxillary palp, ventral **C** fore, middle, and hind legs, ventral **D** forewing, dorsal **E** hind wing, dorsal.

###### Description.

**Male.** Total length 2.1–2.3 mm, 20 antennal segments, scape twice as long as wide. Maxillary palp 5-segmented, terminal segment elongate. Wings brown in alcohol, forewing with venation reduced, cluster of spines basally, and sub-basally, narrow area of sclerotization above basal cluster of spines, jugular lobe present, hindwing thin, venation reduced, narrow band of sclerotization basally. Thorax brown in alcohol, mesoscutellum diamond-shaped with transverse suture, metascutellum triangular. Legs with spur count of 1, 3, 4. ***Genitalia.*** Abdominal segment VII annular, lacking a ventromesal process. Segment VIII complete ventrally, incomplete dorsally; in dorsal view reduced to a pair of elongate lobes, tipped with thickened setae. Segment IX contained within VII and VIII, anteriorly triangular, posteriorly narrowing distally to an acute, sclerotized process which extends past the inferior appendage; in dorsal view, laterally narrow and tapering distally, posterior margin deeply incised; in ventral view similar in shape to that of dorsum. Segment X reduced and membranous. Inferior appendage parallel sided, extending dorsally to downturned apex, bearing heavy setae along ventral margin and apically; in ventral view, wide basally, narrowing distally, thick setae on mesal margin; in dorsal view narrow over length, thickened setae apically. Subgenital plate visible in lateral view as thin basal plate. Phallus extremely long and thin, in dorsal view, apex with narrow, membranous projection on inner margin, thin lateral process originating beyond midlength and extending subapically, ejaculatory duct internal; in lateral view, apex produced into pair of acute hooks.

**Figure 4.**
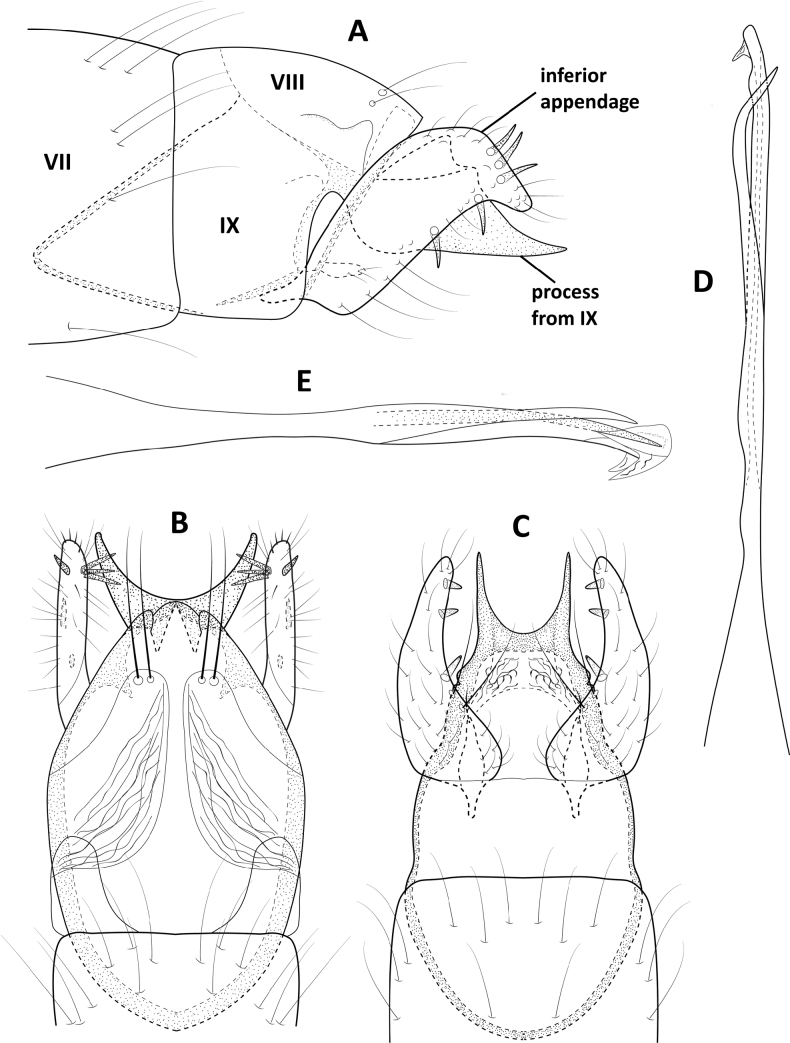
*Angrisanoiabokota* sp. nov., male holotype, genitalia **A** left lateral **B** dorsal **C** ventral **D** phallus, dorsal **E** phallus, left lateral.

###### Distribution.

Panama.

###### Etymology.

This species is named for the indigenous Bokota people who live in Veraguas Province where the species was collected.

###### Remarks.

The body of the adults of *Angrisanoia* have not been illustrated. We have taken the opportunity herein to provide such figures (Fig. 3).

#### Genus *Bredinia* Flint

*Bredinia* is endemic to the Neotropics and is placed in the Stactobiinae. Thomson (2023) listed 17 species from the Neotropics, four of which had been recorded from Panama (Armitage et al. 2016, 2018). Herein we describe and illustrate a new species to Panama’s fauna.

##### ﻿ Bredinia paraespinosa sp. nov.

Taxon classificationAnimaliaTrichopteraHydroptilidae

D1BAC07E-F4E9-5246-B2A6-BAD44BF08978

https://zoobank.org/AF52F1C4-E38D-4CAE-8FAE-93996558E9E0

Fig. 5

###### Type locality.

**Panama: Veraguas Province**: Cuenca 097; Santa Fe District; Santa Fe NP; Río Piedra de Moler; PSPSCB-PNSF-C097-2017-012; 8.56553°N, 81.18817°W; 340 m a.s.l.

###### Type material.

***Holotype*: male, Panama: Veraguas Province**: Cuenca 097; Santa Fe District; Santa Fe NP; Río Piedra de Moler; PSPSCB-PNSF-C097-2017-012; 8.56553°N, 81.18817°W; 340 m a.s.l.; UV light trap; A. Cornejo, T. Ríos, E. Álvarez, C. Nieto, leg.; 20.iv.2017; MIUP-003-T-2023 (in alcohol).

###### Diagnosis.

This species is similar to *B.espinosa* Harris, Holzenthal & Flint, from Brazil, Ecuador, French Guiana, and Venezuela, both having prominent stout, spine-like setae arising from the sides of segment VIII. *Brediniaparaespinosa* sp. nov. differs from this species in the elongate anterior apodemes from the lobes of segment VIII and the structure of the phallus which has the sides bearing an elongate apical spine.

**Figure 5.**
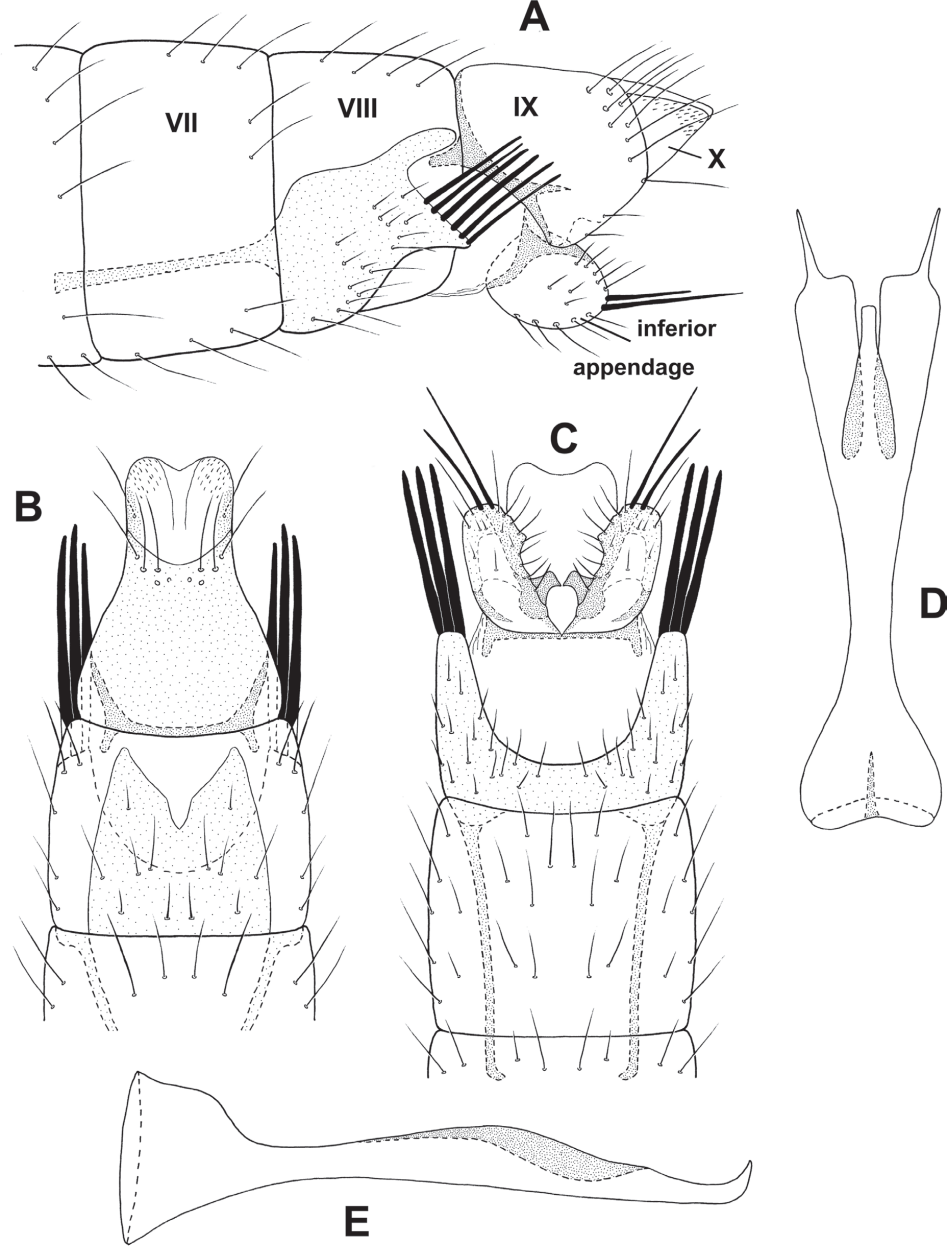
*Brediniaparaespinosa* sp. nov., male holotype, genitalia **A** left lateral **B** dorsal **C** ventral **D** phallus, dorsal **E** phallus, left lateral.

###### Description.

**Male.** Total length 1.5 mm, 17 antennal segments, wings and body brown in alcohol. **G*enitalia.*** Abdominal segment VII annular, lacking a ventromesal process. Segment VIII annular, lateral lobe bearing prominent, spinelike setae, anterior margin produced into elongate apodeme, projecting into segment VI; in dorsal view with central plate which appears to be part of the lateral lobe; in ventral view, deeply incised mesally, lateral lobes with elongate posterior spines. Segment IX narrowing ventrally; in dorsal view tapering distally to emarginated apex; ventrally reduced and membraneous. Segment X triangular in lateral view, thin with posterior emargination in dorsal and ventral views. Inferior appendage circular in lateral view, bearing pair of elongate, stout setae distally; in ventral view spatulate, inner margin diverging, with hooklike sclerite basally. Subgenital plate thin in lateral view, bifid apically; in dorsal and ventral views a narrow band, with extended lateral margins. Phallus tubular, posteriorly widening with lateral apices narrowed to acute spine, mesally diverging with ejaculatory duct protruding; in lateral view wide basally, tapering distally to upturned apex.

###### Distribution.

Panama.

###### Etymology.

This species name *paraespinosa* (spiny-like) derives from Spanish, referring to its spiny resemblance to *Brediniaespinosa*. The name is a noun in the nominative singular standing in apposition.

#### Genus *Cerasmatrichia* Flint, Harris & Botosaneanu

A member of the Leucotrichiinae, *Cerasmatrichia* is endemic to the Neotropics, ranging from Costa Rica south to Peru, east to Trinidad and throughout the Lesser Antilles (Holzenthal and Calor 2017). Male members of the genus may display sexual dimorphism in scaly patches found on the wings and modified maxillary palps on the head (Flint et al. 1994). Of the 11 species in the genus, five species are known from Panama, four of which were described from there (Armitage et al. 2016; Harris and Armitage 2019; Armitage and Harris 2020; Harris et al. 2023). Here we describe and figure two additional species in the Neotropical fauna of Panama, one of which displays a modified wing in the male.

##### ﻿ Cerasmatrichia garfioza sp. nov.

Taxon classificationAnimaliaTrichopteraHydroptilidae

C16775F8-D7F7-5604-94BB-4FB84894F396

https://zoobank.org/0C98338C-A8A6-4F56-9DAE-6D632B114D20

Fig. 6

###### Type locality.

**Panama: Veraguas Province**: Cuenca 132; Veraguas Province; Cuenca 132; Santa Fe District; Santa Fe NP; Quebrada Mulabá; PSPSCB-PNSF-C132-2017-008; 8.51706°N, 81.12140°W; 770 m a.s.l.

###### Type specimen.

***Holotype*, male. Panama: Veraguas Province**: Cuenca 132; Veraguas Province; Cuenca 132; Santa Fe District; Santa Fe NP; Quebrada Mulabá; PSPSCB-PNSF-C132-2017-008; 8.51706°N, 81.12140°W; 770 m a.s.l.; Malaise trap; T. Ríos, E. Álvarez, C. Nieto, leg.; 19–23.iv.2017; MIUP-004-T-2023 (in alcohol).

###### Other material examined.

**Panama: Chiriqui Province** • 1 male, Cuenca 108; Boquete District; Quebrada Jaramillo; off Jaramillo Alto Rd.; Collier property; 8.76320°N, 82.41383°W; 1259 m a.s.l.; Malaise trap; K. Collier, leg.; 8–12.v.2018; MUPADI.

###### Diagnosis.

This species is similar to *C.akanthos* Armitage & Harris, from Panama, as well as *C.hidala* Oláh & Johanson from Peru, but as noted in the descriptions of these species, they do not fit well within the generic limits of *Cerasmatrichia*. As with both these species, *C.garfioza* sp. nov. has three ocelli, the leg spurs are 1,3,4, and the phallus is tubular, all characteristic of the genus. In common with *C.hidala*, the forewing of this new species has a small scaly area. *Cerasmatrichiagarfioza* sp. nov. is easily identified by the sclerotized hook-like process on abdominal segment IX.

**Figure 6.**
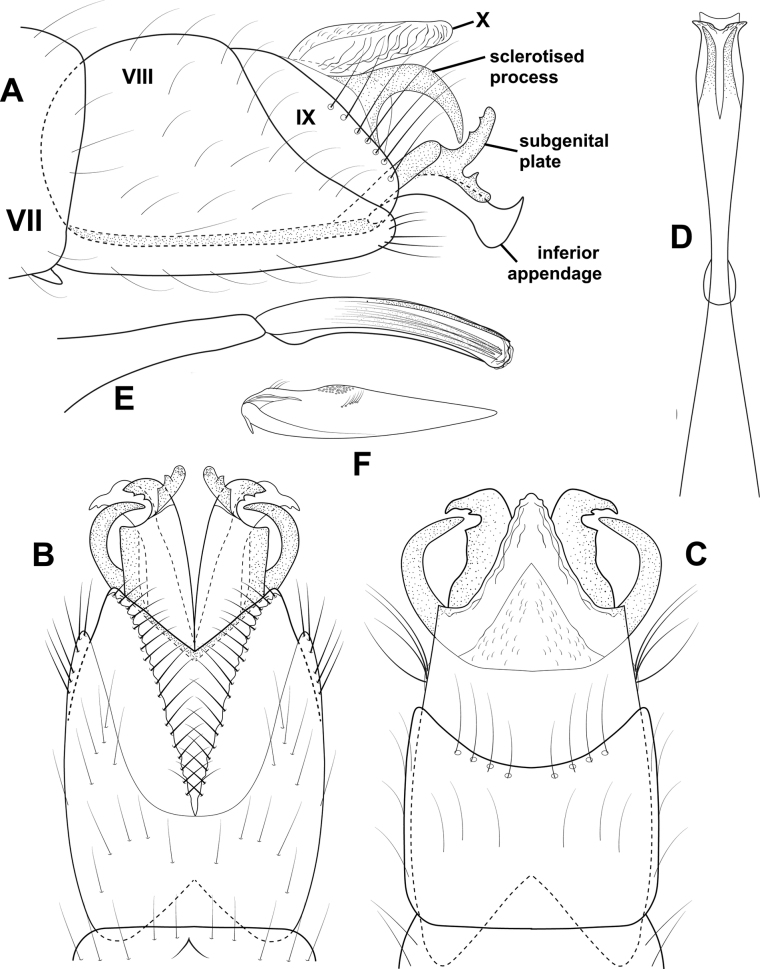
*Cerasmatrichiagarfioza* sp. nov., male holotype, genitalia **A** left lateral **B** ventral **C** dorsal **D** phallus, dorsal **E** phallus, left lateral **F** forewing, dorsal.

###### Description.

**Male.** Total length 2.2–2.4 mm, head unmodified, antennae with 21 segments, scape and pedicel each twice as long as proximal flagellomeres, forewings dark brown with pair of white bands at midlength and apex, small scaly patch along dorsal margin, body brown in alcohol. **G*enitalia***. Abdominal segment VII annular with small ventromesal process. Segment VIII tapering ventrad on posterior margin; in ventral view, broadly and deeply emarginate; in dorsal view squarish, slightly emarginated posteriorly. Segment IX somewhat rectanguloid, anteriorly tapering to rounded ventral point, posteriorly divided into two sections, anterior section tapering posteriorly, bearing stout setae on margin, posterior section truncate distally, bearing elongate hooklike sclerotized process posterodorsally; in dorsal view elongate, shallowly emarginate posteriorly, deeply emarginate anteriorly; in ventral view deeply incised posteriorly, with row of elongate setae on mesal margin. Segment X shelflike in lateral view; in dorsal view triangular, membranous distally. Inferior appendage thin and elongate, widening distally and upturned; in ventral view narrow over length, curving outward distally to acute points. Subgenital plate narrow in lateral view, divided posteriorly into elongate dorsal process and shorter ventral process; in ventral view, lower portion rectangular, abruptly narrowing distally to pair of short processes. Phallus tubular, in lateral view narrowing near midlength, apical portion cylindrical with cluster of elongate sclerotized rods; in dorsal view cylindrical apically, pair of diverging, subapical rods.

###### Distribution.

Panama.

###### Etymology.

The species name ‘*garfioza*’ (hook) derives from Spanish, referring to the posterodorsal hooklike process on segment IX. The name is a noun in the nominative singular standing in apposition.

##### ﻿ Cerasmatrichia veraguasensis sp. nov.

Taxon classificationAnimaliaTrichopteraHydroptilidae

DB1C6A6D-FDEF-5D0D-997D-445F4D9BBFD1

https://zoobank.org/BCA59CC3-DBFC-4784-A74A-CFF47C46F8B5

Fig. 7

###### Type locality.

**Panama: Veraguas Province**: Cuenca 132; Santa Fe District; Santa Fe NP; Quebrada Primer Brazo Mulabá; PSPSCB-PNSF-C132-2017-007; 8.52577°N, 81.13045°W; 623 m a.s.l.

###### Type specimen.

***Holotype*: male, Panama: Veraguas Province**; Cuenca 132; Santa Fe District; Santa Fe NP; Quebrada Primer Brazo Mulabá; PSPSCB-PNSF-C132-2017-007; 8.52577°N, 81.13045°W; 623 m a.s.l.; Malaise trap; 19–23.iv.2017; A. Cornejo, T. Ríos, E. Álvarez, C. Nieto, leg.; MIUP-005-T-2023 (in alcohol). ***Paratype***: Same as for holotype; 1 male; MUPADI (in alcohol).

###### Diagnosis.

This species is similar to *C.akanthos* Armitage & Harris, from Panama and *C.garfioza* sp. nov., both of which have an elongate posterior process from segment IX. The new species is easily identified by the sclerotized process from abdominal segment VIII, the absence of the inferior appendage, and the lack of a ventromesal process on abdominal segment VIII.

**Figure 7.**
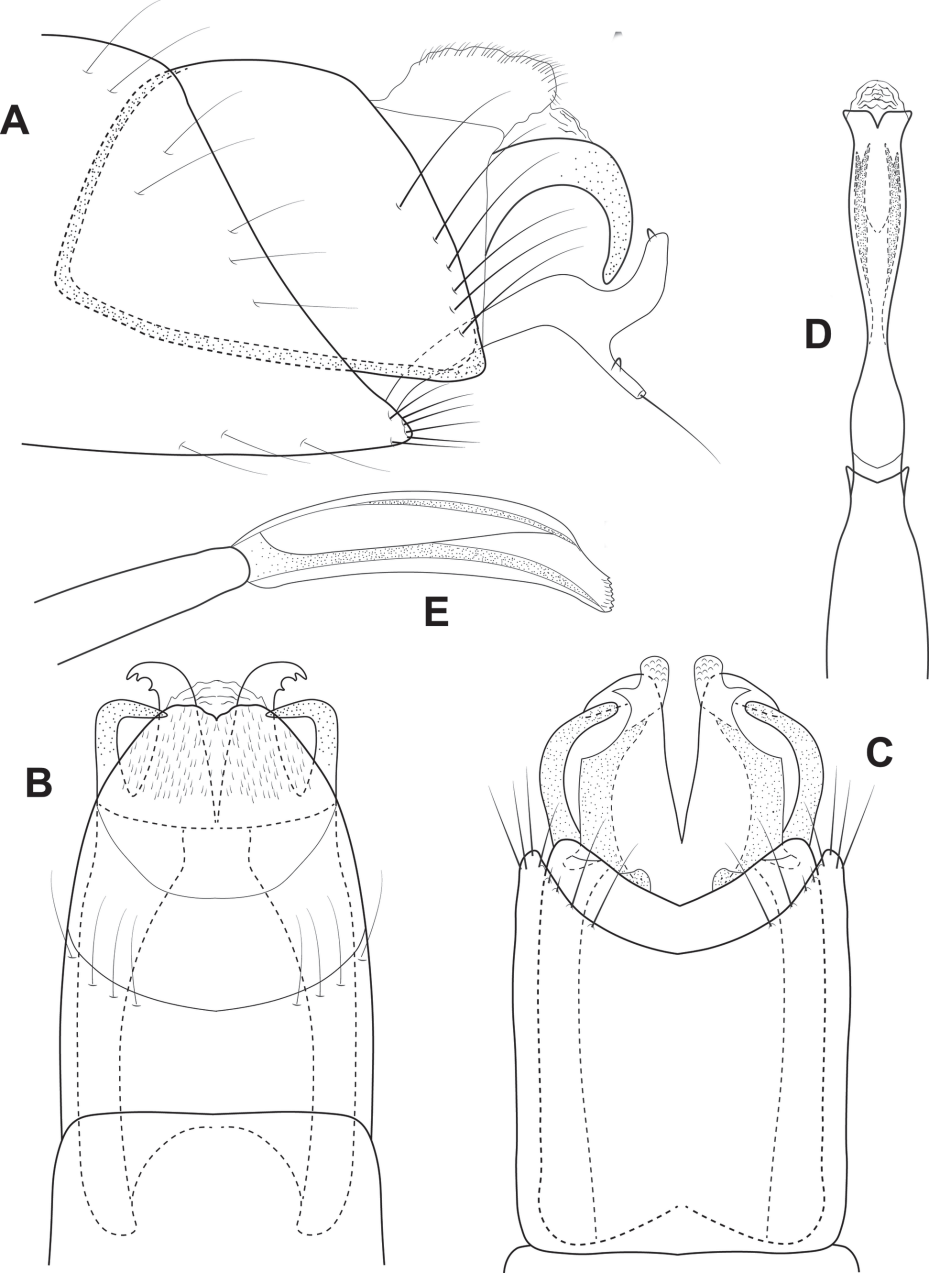
*Cerasmatrichiaveraguasensis* sp. nov., male holotype, genitalia **A** left lateral **B** dorsal **C** ventral **D** phallus, dorsal **E** phallus apex, left lateral.

###### Description.

**Male.** Total length 1.8 mm, head unmodified, antennae with 21 segments, scape and pedicel twice as long as proximal flagellomeres, forewings dark brown with white horizontal bands in middle, body brown in alcohol. **G*enitalia*.** Abdominal segment VII annular without ventromesal process. Segment VIII tapering posteroventrally; in ventral view quadrate, broadly emarginate posteriorly, clump of stout setae on lateral margins; in dorsal view, narrow, posterior margin emarginate. Segment IX somewhat rectanguloid, anteriorly truncate, tapering ventrad, posterior margin tapering ventrad, prominent hooklike sclerotized process posterodorsally from truncate extension of IX; in dorsal view elongate, anteriorly deeply emarginate, posteriorly divided into two sections, lower section thin and bandlike, upper section roundly tapering distally, slightly incised mesally on posterior margin, lateral posterior processes curving sharply inward apically; in ventral view rectangular, slightly incised anteriorly and posteriorly, narrow posteriorly, lateral processes gently curving inward distally. Segment X bulbous laterally, setose on dorsal surface, dorsally triangular, membranous distally. Inferior appendage lacking, although the process from segment VIII may constitute an inferior appendage. Subgenital plate in lateral view bifid, upper arm thicker and longer than lower, each bearing short spikes, in dorsal view mesally divided into triangular processes which curve outward distally, with serrate outer margins; in ventral view divided mesally, abruptly tapering subapically, apex rounded with lateral spike. Phallus tubular, in lateral view slightly narrowing near midlength, apical portion cylindrical with pair of elongate sclerotized rods; in dorsal view cylindrical apically, narrowing laterally, apically with incised flange, pair of subapical rods which slightly diverge apically.

###### Distribution.

Panama.

###### Etymology.

This species is named for the Veraguas Province, where the species was collected.

#### Genus *Costatrichia* Mosely

*Costatrichia* is a leucotrichiine genus endemic to the Neotropics and distributed from Mexico south to Argentina. This genus is currently represented by 20 species (Thomson 2023), seven of which are found in Panama (Holzenthal and Calor 2017; Thomson and Armitage 2018; Harris and Armitage 2019). Here we add one new species to Panama’s fauna.

##### ﻿ Costatrichia calovebora sp. nov.

Taxon classificationAnimaliaTrichopteraHydroptilidae

7B1F94BB-7BF6-5077-9050-4868D5A8F64E

https://zoobank.org/680A263F-74EE-410E-A4D4-29FF51C1D1BA

Fig. 8

###### Type locality.

**Panama: Veraguas Province**: Cuenca 097; Santa Fe District; Santa Fe NP; Río Calovébora; PSPSCB-PNSF-C-097-2017-005; 8.54318°N, 81.16398°W; 536 m a.s.l.

###### Type specimen.

***Holotype*: male, Panama: Veraguas Province**: Cuenca 097; Santa Fe District; Santa Fe NP; Río Calovébora; PSPSCB-PNSF-C-097-2017-005; 8.54318°N, 81.16398°W; 536 m a.s.l.; Malaise trap; T. Ríos, E. Álvarez, C. Nieto, leg.; 19–23.iv.2017; MIUP-006-T-2023 (in alcohol).

###### Diagnosis.

This species is most similar to three other species (*C.tripartita* Flint from Costa Rica and Panama, and *C.carara* Holzenthal & Harris and *C.venezuelensis* Flint, both reported from Costa Rica; Holzenthal and Calor 2017), all of which have a tripartite inferior appendage. It differs from these species in the structure of the inferior appendage, the lack of mesoventral horns on abdominal segment VIII, which are found in *C.carara and C.venezuelensis*, and in the poorly formed spines of the phallus.

###### Description.

**Male.** Total length 3.5 mm, head missing, forewing with elongate costal bulla, body and wings brown in alcohol. ***Genitalia*.** Abdominal segment VII annular with prominent ventromesal process. Segment VIII triangular, reduced dorsally; in ventral view elongate, posteriorly with deep incision. Segment IX generally quadrate, truncate anteriorly, posterior margin slanted, with dorsal knob, laterally with seta-bearing process; in ventral view narrow; in dorsal view enclosed within VIII, anteriorly with shallow emargination, lateral margins sclerotized. Segment X reduced to a short shelf in lateral view; dorsally rounded and membranous. Inferior appendage divided into three elongate processes, dorsalmost process thin and extending ¾ length of mesal and ventralmost processes, narrowing distally, ventralmost process rectangular, truncate distally, mesal process widening at midlength, then abruptly tapering to acute apex; in ventral and dorsal view mesal process tapering apically, slightly longer than ventralmost process which is club-like apically, lateral process about half length of others and tapering distally. Phallus in lateral view wide basally and subapically, narrow at midlength complex which bears sclerotized window and basal loop, subapical spine which is poorly formed and blunt apically; in dorsal view lacking acute spines, subapically divided into pair of elongate flattened plates.

**Figure 8.**
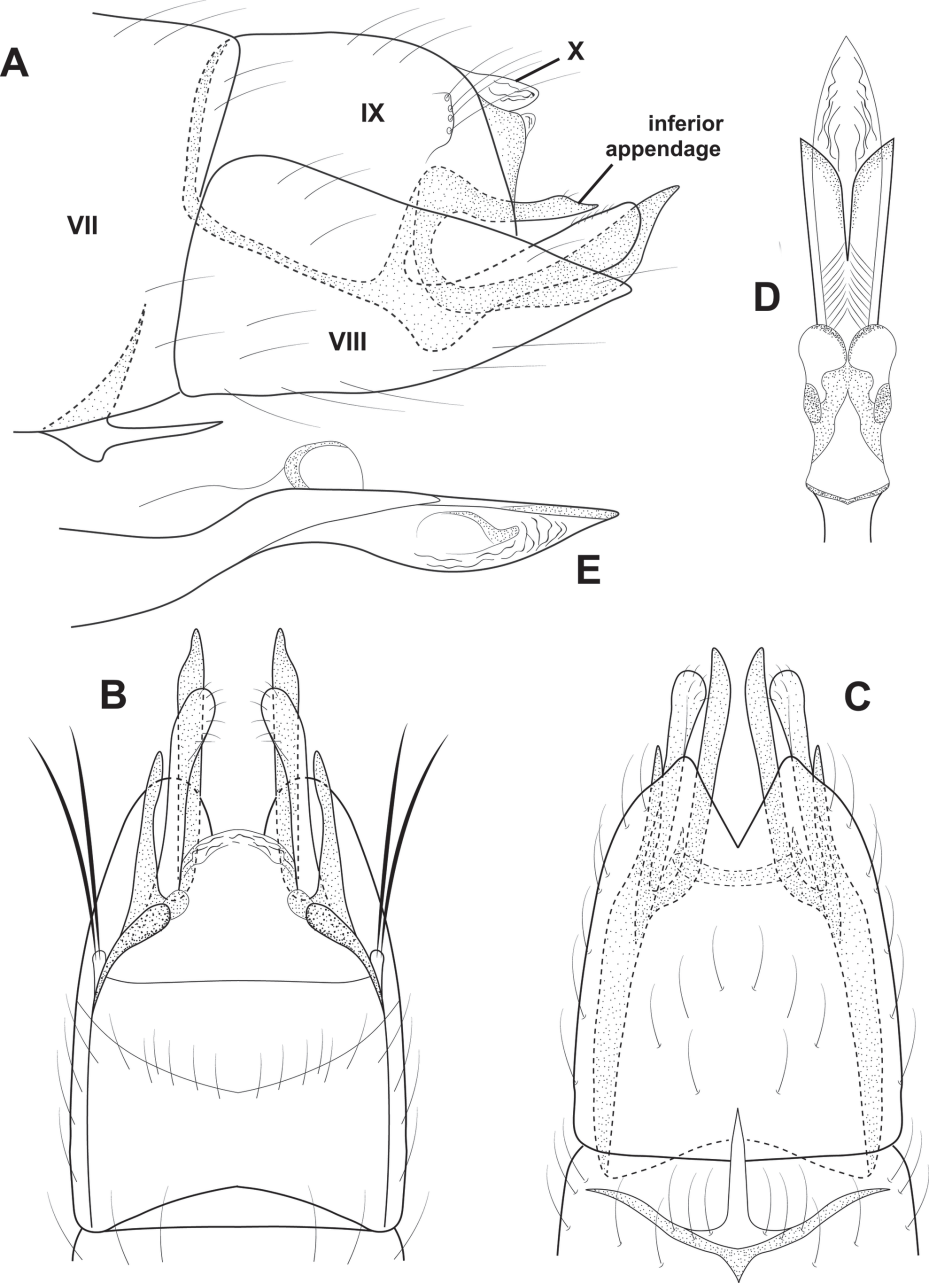
*Costatrichiacalovebora* sp. nov., male holotype, genitalia **A** left lateral **B** dorsal **C** ventral **D** phallus apex, dorsal **E** phallus apex, left lateral.

###### Distribution.

Panama.

###### Etymology.

This species is named for the Río Calovébora watershed, where the species was collected.

#### Genus *Metrichia* Ross

The genus *Metrichia* (Trichoptera: Hydroptilidae: Hydroptilinae: Ochrotrichiini) is represented by 144 species (Thomson 2023) endemic to the New World and distributed in North, Central, and South America (Santos et al. 2016; Harris and Armitage 2015, 2019; Thomson and Armitage 2018;Armitage and Harris 2020). Previously, 40 species were recorded from Panama (Flint 1972; Bueno-Soria and Santiago-Fragoso 2002; Bueno-Soria and Holzenthal 2003; Armitage et al. 2015, 2016, 2022a; Harris and Armitage 2019; Armitage and Harris 2020, 2023; Thomson and Armitage 2021; Harris et al. 2023). Herein we describe and figure six new species.

##### ﻿ Metrichia calovebora sp. nov.

Taxon classificationAnimaliaTrichopteraHydroptilidae

26042750-50E9-518A-9FF7-F8A53FF80953

https://zoobank.org/346E7B0D-53FF-4AF8-8B39-44D23F5B3903

Fig. 9

###### Type locality.

**Panama: Veraguas Province**: Cuenca 097; Santa Fe District; Santa Fe NP; Río Calovébora; PSPSCB-NPSF-C-097-2017-005; 8.54318°N, 81.16398°W; 536 m a.s.l.

###### Type specimen.

***Holotype*: male, Panama: Veraguas Province**: Cuenca 097; Santa Fe District; Santa Fe NP; Río Calovébora; PSPSCB-NPSF-C-097-2017-005; 8.54318°N, 81.16398°W; 536 m a.s.l.; Malaise trap; T. Ríos, E. Álvarez, C. Nieto, leg.; 19–23.iv.2017; MIUP-007-T-2023 (in alcohol). ***Paratypes***: Same as for holotype; 2 males; MUPADI (in alcohol).

###### Diagnosis.

This species with the posteromesally incised inferior appendage is similar to *M.palida* Bueno-Soria & Santiago-Fragoso and *M.thomsonae* Harris & Armitage both of which occur in Panama. However, the phallus is more similar to *M.cafetalera* Botosoneanu from Cuba, which also has a pair of prominent apical hooked spines; however, the phallus of *M.cafetalera* lacks the lateral process seen in *M.calovebora* sp. nov.. Also, the inferior appendage of *M.cafetalera* differs in the ventral position of the posterior incision.

**Figure 9.**
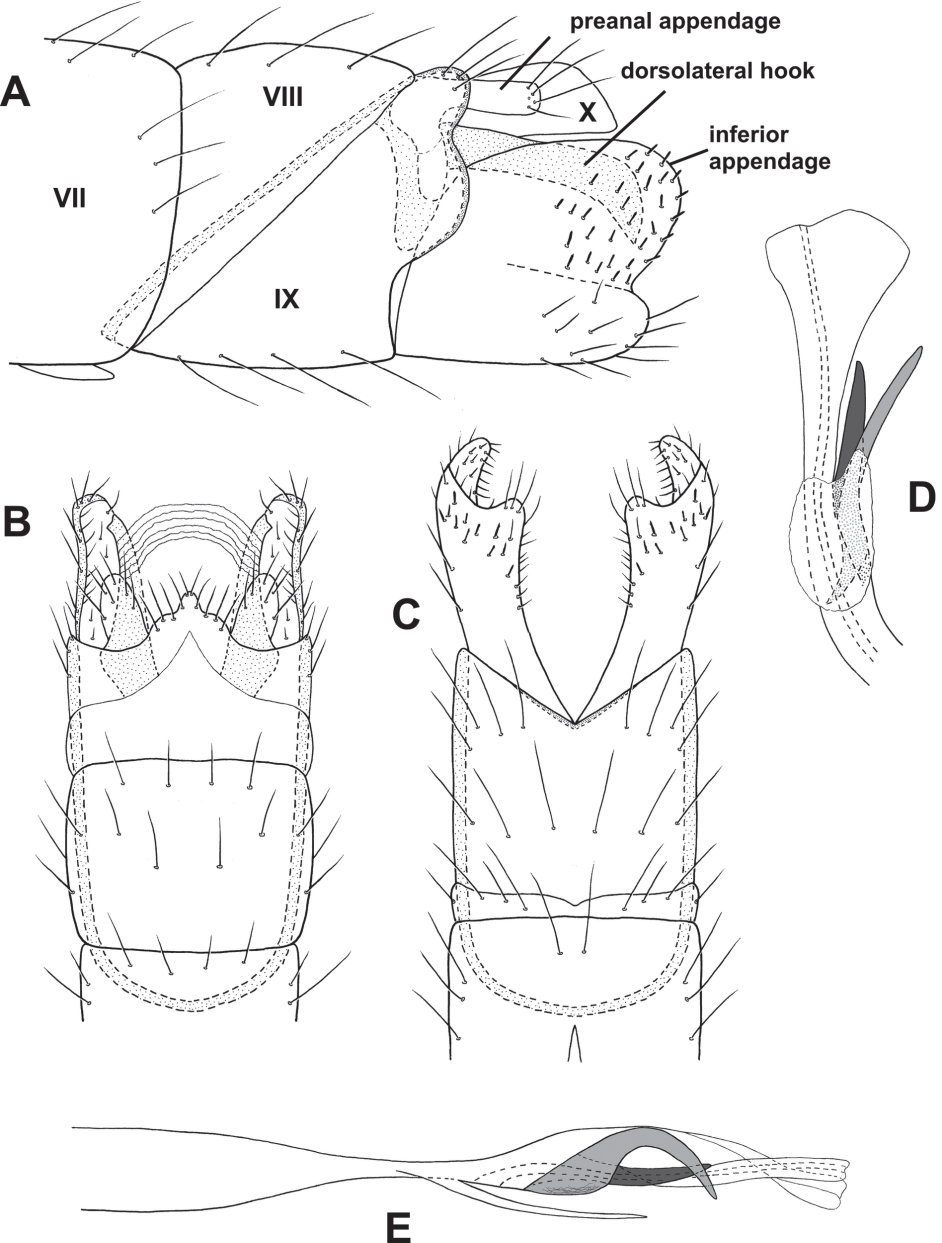
*Metrichiacalovebora* sp. nov., male holotype, genitalia **A** left lateral **B** dorsal **C** ventral **D** phallus apex, left lateral **E** phallus, dorsal.

###### Description.

**Male.** Total length 2.1–2.3 mm, 18 antennal segments, forewings with patch of scales on anterior portion, body brown in alcohol. Abdominal segment V with small pair of rounded sacs on dorsum. **G*enitalia*.** Segment VII annular with short ventromesal process. Segment VIII triangular, tapering ventrally; in ventral view very short, slight incision mesally on posterior margin, anterior margin rounded; in dorsal view truncate. Segment IX triangular, tapering anteriorly into segment VII, posterior margin sinuate; in ventral view incised mesally on posterior margin; anterior margin rounded; in dorsal view posterior margin expanded mesally. Preanal appendage (cercus) rectangular in lateral view, dorsally ovate. Dorsolateral hook in lateral view narrow over length, tapering apically and curving ventrad; in dorsal view wide basally, tapering to acute apices, curving on outer margin with subapical spike. Segment X shelf-like in lateral view, tapering distally; in dorsal view broadly rounded, membranous apically. Inferior appendage in lateral view incised posteroventrally, dorsal lobe larger than ventral lobe, dorsal and ventral margins nearly parallel-sided; in ventral view narrow over length, subapically abruptly narrowing on inner surface, diverging basally and curved on mesal margin; in dorsal view thin and rectanguloid. Phallus in dorsal view narrowing at midlength, where originates a lateral process, distal portion with pair of large, hooked spines, which appear to be bifid, subapical in position; in lateral view, outer subapical spine more curved than inner spine which appears to originate from base of outer spine, apex expanded and plate-like.

###### Distribution.

Panama.

###### Etymology.

This species is named for the Río Calovébora where the species was collected.

##### ﻿ Metrichia cascada sp. nov.

Taxon classificationAnimaliaTrichopteraHydroptilidae

B10FC285-4AC7-5175-9DE9-DAFFE554FF56

https://zoobank.org/F7F609A6-F108-48BD-A3EE-1E17238D4002

Fig. 10

###### Type locality.

**Panama: Chiriqui Province**: Cuenca 108; Boquete District; Volcán Barú NP; Río del Guayabo; PSPSCB-PNVB-C108-2017-018; 8.84939°N, 82.49349°W; 1947 m a.s.l.

###### Type specimen.

***Holotype*: male, Panama: Chiriqui Province**: Cuenca 108; Boquete District; Volcán Barú NP; Río del Guayabo; PSPSCB-PNVB-C108-2017-018; 8.84939°N, 82.49349°W; 1947 m a.s.l.; Malaise trap; E. Álvarez, E. Pérez, T. Ríos; 5–8.vi.2017; MIUP-008-T-2023 (in alcohol).

###### Diagnosis.

Similar in many respects to both *M.pakitza* Flint & Bueno-Soria and *M.diosa* Flint & Bueno-Soria, both from Peru, *M.cascada* sp. nov. is distinguished from these species by the sharply narrowed posterior of the inferior appendage in lateral view, which is also deeply incised posteroventrally. There is also a spinose projection from the basal anterior margin and a knob-like process from the posterior margin of the inferior appendage when viewed in dorsal or ventral views in *M.cascada* sp. nov., which is not seen in the related species.

**Figure 10.**
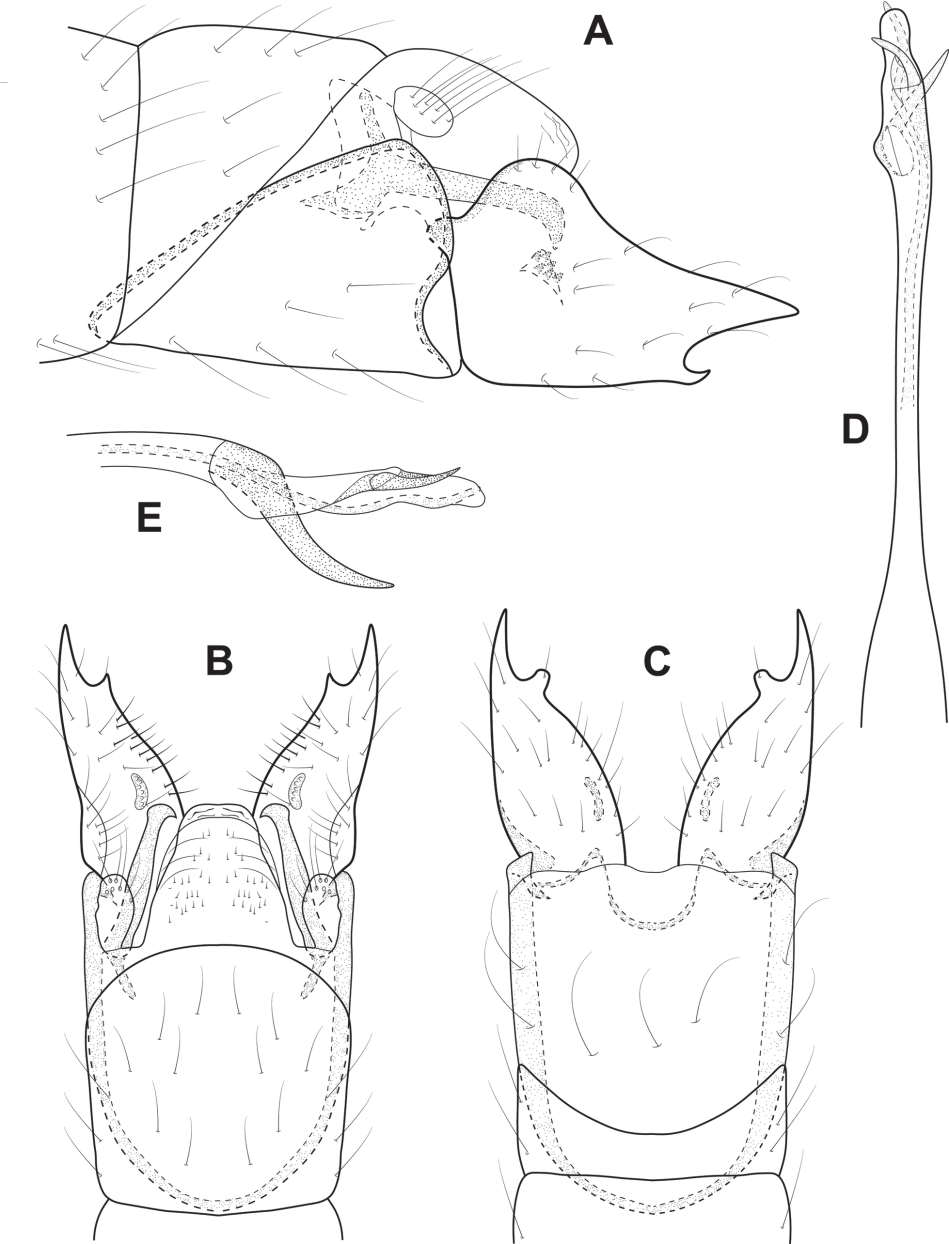
*Metrichiacascada* sp. nov., male holotype, genitalia **A** left lateral **B** dorsal **C** ventral **D** phallus, dorsal **E** phallus apex, left lateral.

###### Description.

**Male.** Total length 1.9 mm, antenna short with 17 segments, middle segments flattened and widening, wings and body brown in alcohol, abdominal terga without modifications. **G*enitalia*.** Abdominal segment VII annular without ventromesal process. Segment VIII triangular, tapering ventrally; in ventral view very short, broad emargination posteriorly; in dorsal view truncate. Segment IX triangular, tapering anteriorly into segment VII, posterior margin sinuate; in ventral view slightly incised mesally on posterior margin, anterior margin rounded; narrow in dorsal view. Preanal appendage (cercus) ovate laterally and dorsally. Dorsolateral hook in lateral view narrow over length, tapering apically and curving ventrad; in dorsal view narrow over length, apices rounded and tapering inward. Segment X lobate in lateral view, rounded distally; in dorsal view broadly triangular, apex truncate and membranous. Inferior appendage in lateral view wide basally and rounded, tapering distally, incised posteroventrally, upper portion long and pointed apically, lower portion short and acute, process with numerous dark peg-like setae from basal inner margin; in ventral view wide basally, tapering distally, subapical knob on inner margin; in dorsal view wide basally, tapering distally, subapical spike on inner margin, renal-shaped process bearing short pegs on inner margin near base. Phallus in dorsal view wide basally, narrowing apically, distal portion with pair of spines, subapical spine larger than apical spine, and straight, apical spine short and curved; in lateral view, subapical spine twice as long and thicker than apical spine, curving slightly under phallic shaft, apical spine short and dorsal in position.

###### Distribution.

Panama.

###### Etymology.

The species name *cascada* (cascade or waterfall) derives from Spanish, referring to the cascade on the Río del Guayabo where the species was collected.

##### ﻿ Metrichia chiriquiensis sp. nov.

Taxon classificationAnimaliaTrichopteraHydroptilidae

18C22E3E-FACF-5059-B957-2710AA5C6423

https://zoobank.org/D15E6B2B-E3FD-4FB2-828A-5D6A952AC142

Fig. 11

###### Type locality.

**Panama: Chiriqui Province**: Cuenca 108; Boquete District; Volcán Barú NP; Río del Guayabo; PSPSCB-PNVB-C108-2017-018; 8.84939°N, 82.49349°W; 1947 m a.s.l.

###### Type specimen.

***Holotype*: male, Panama: Chiriqui Province**: Cuenca 108; Boquete District; Volcán Barú NP; Río del Guayabo; PSPSCB-PNVB-C108-2017-018; 8.84939°N, 82.49349°W; 1947 m a.s.l.; Malaise trap; E. Álvarez, E. Pérez, T. Ríos; 5–8.vi.2017; MIUP-009-T-2023 (in alcohol).

###### Diagnosis.

This species with the ventrally elongate inferior appendage is similar to *M.calla* Thomson & Armitage, *M.plax* Thomson & Armitage, and *M.sesquipedalis* Bueno-Soria & Holzenthal, all of which occur in Panama. The new species is recognized by the deeply incised posterior margin of the inferior appendage, with the dorsal process nearly half as long as the ventral process, which in *M.calla* and *M.plax* is short, and absent in *M.sesquipedalis*; additionally, the inferior appendage in *M.chiriquiensis* sp. nov. is prominently serrate on the ventral margin. Also, the abdominal terga of *M.chiriquiensis* sp. nov. lack modifications as does *M.sesquipedalis*, while those of *M.calla* have long hair clusters and *M.plax* has elongate lobes.

**Figure 11.**
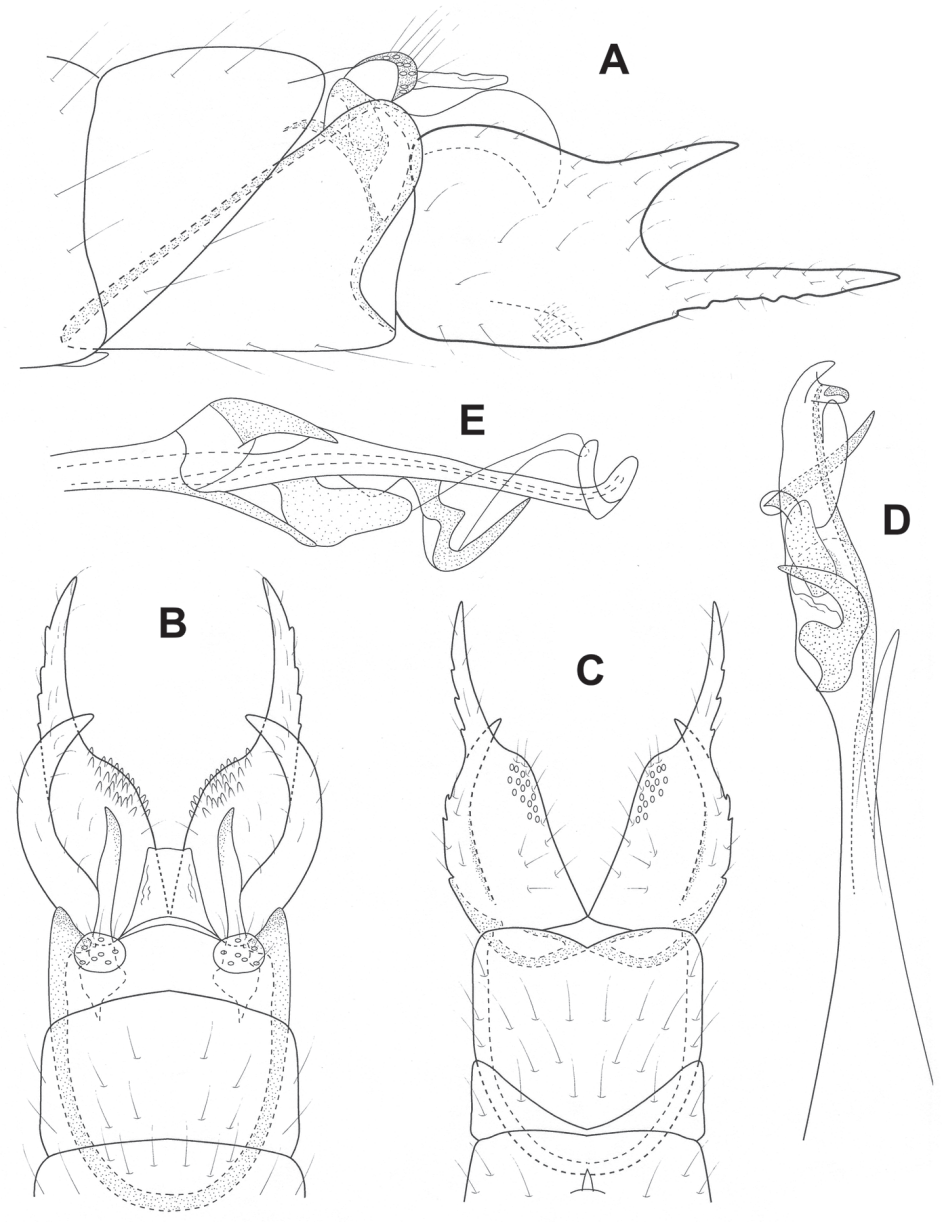
*Metrichiachiriquiensis* sp. nov., male holotype, genitalia **A** left lateral **B** dorsal **C** ventral **D** phallus, dorsal **E** phallus apex, left lateral.

###### Description.

**Male.** Total length 2.8 mm, 20 antennal segments, middle segments widening and flat, wings and body brown in alcohol. Abdominal terga without modifications. ***Genitalia*.** Segment VII annular with short ventromesal process. Segment VIII triangular, tapering ventrally; in ventral view very narrow, deep mesal incision; in dorsal view truncate. Segment IX triangular, tapering anteriorly into segment VII, posterior margin sinuate; in ventral view slightly incised mesally on posterior margin; anterior margin rounded; in dorsal view narrow. Preanal appendage (cercus) ovate in lateral view, dorsally circular. Dorsolateral hook in lateral view thin basally, then widening subapically, tapering distally, and curving ventrad; in dorsal view narrow, tapering to acute apices, sinuate on outer margins. Segment X in lateral view short, thin, and membranous; in dorsal view short, tapering distally to truncate apex. Inferior appendage in lateral view deeply incised posteromesally forming two thin elongate processes, dorsal process about half length of ventral process, which is serrate on ventral margin; in ventral view lower process wide basally, narrowing subapically, diverging basally with outer edge serrate; in dorsal view upper process thin over length and strongly curving mesad. Phallus in dorsal view narrowing at midlength, where originates a lateral process, distal portion with pair of large spines, lower spine curved, upper spine sinuate; in lateral view, lower spine dorsal in position and about half length of upper sinuate spine, apex with curved sclerotized process.

###### Distribution.

Panama.

###### Etymology.

This species is named for Chiriqui Province where the species was collected.

##### ﻿ Metrichia escobilla sp. nov.

Taxon classificationAnimaliaTrichopteraHydroptilidae

9D01A624-9EBE-5054-9CBB-10C9DB9B6221

https://zoobank.org/368B4424-5833-4E39-937F-AAF99D1E183D

Fig. 12

###### Type locality.

**Panama: Veraguas Province**: Cuenca 097; Santa Fe District; Santa Fe NP; Río Calovébora; PSPSCB-PNSF-C097-2017-005; 8.54318°N, 81.16398°W; 536 m a.s.l.

###### Type specimen.

***Holotype*: male, Panama: Veraguas Province**: Cuenca 097; Santa Fe District; Santa Fe NP; Río Calovébora; PSPSCB-PNSF-C097-2017-005; 8.54318°N, 81.16398°W; 536 m a.s.l.; UV light trap; T. Ríos, E. Álvarez, C. Nieto, leg.; 21.iv.2017; MIUP-010-T-2023 (in alcohol).

###### Diagnosis.

This species shares a number of character states with *M.angulosa* Bueno-Soria & Holzenthal, which occurs in Costa Rica and Panama. Both species have a similarly shaped inferior appendage, with dentate posterior margin, and both have apical spines on the phallus. However, while *M.angulosa* has prominent reniform pouches on the dorsum of abdominal segment V, the abdomen of *M.escobilla* sp. nov. lacks abdominal modifications. As well, the new species has both phallic spines pointed, rather than one truncate as in *M.angulosa*, the dentation on the posterior margin of the inferior appendage is more pronounced, and the brush-like apex of the dorsolateral hook is unique to *M.escobilla* sp. nov..

**Figure 12.**
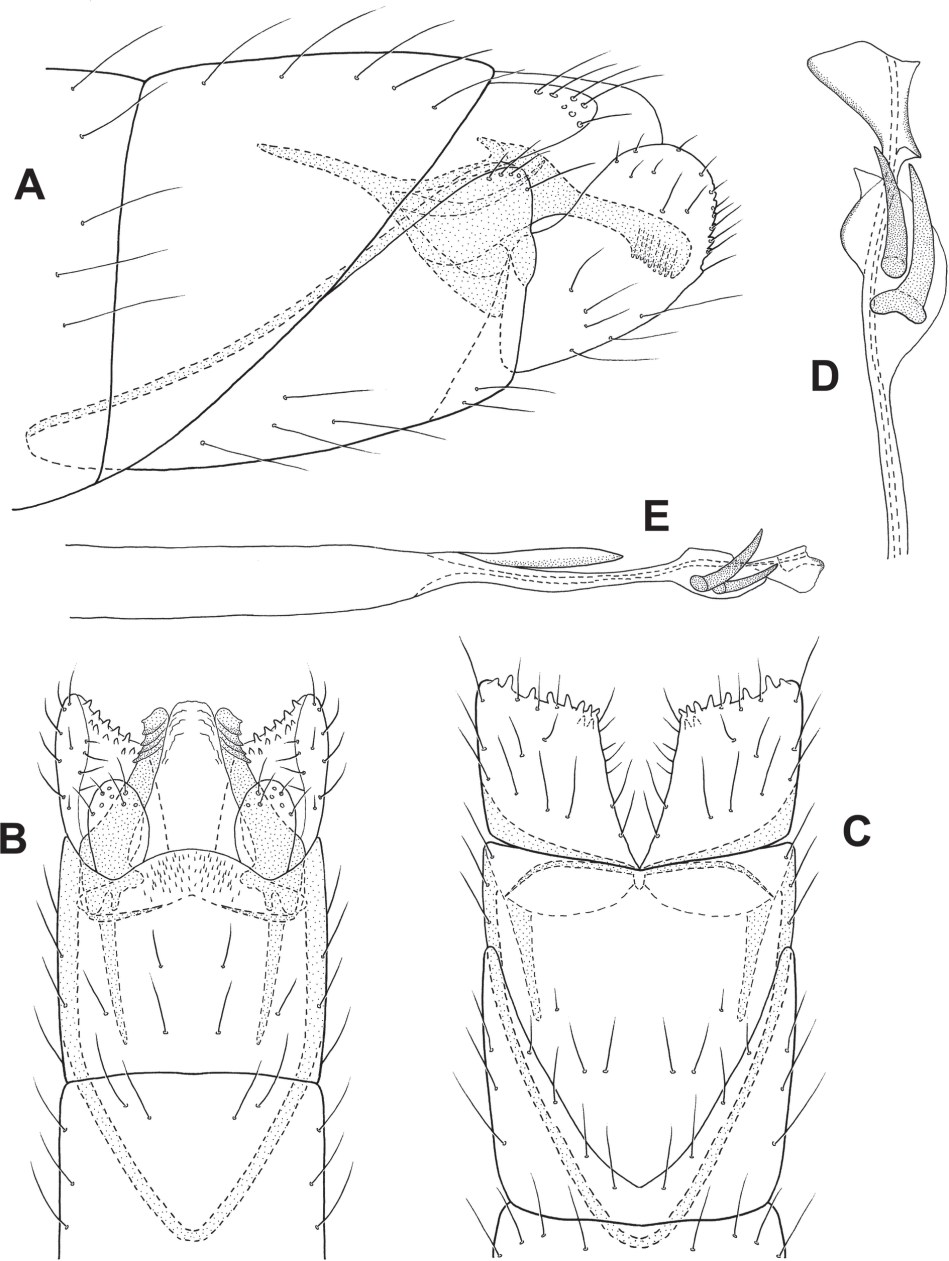
*Metrichiaescobilla* sp. nov., male holotype, genitalia **A** left lateral **B** dorsal **C** ventral **D** phallus apex, left lateral **E** phallus, dorsal.

###### Description.

**Male.** Total length 1.6 mm, 18 antennal segments, wings and body brown in alcohol, abdominal terga without modifications. ***Genitalia*.** Abdominal segment VII annular without short ventromesal process. Segment VIII triangular, tapering ventrally; in ventral view deeply incised mesally; in dorsal view quadrate. Segment IX triangular, truncate posteriorly, tapering anteriorly into segment VII. Preanal appendage (cercus) oval in lateral and dorsal views. Dorsolateral hook in lateral view wide basally, converging near midlength, apex serrate ventrally; in dorsal view wide basally, tapering to serrate margins at apices. Segment X lobate; in dorsal view triangular, membranous apically. Inferior appendage in lateral view narrow at base then widening dorsally, rounded apically; in dorsal and ventral view quadrate, diverging basally, posterior margins dentate. Phallus very long, extending into segment V; in dorsal view wide basally, narrowing posteriorly, pair of spines apically, subapical spine longer than apical, lateral process originating at midlength; in lateral view, subapical spine slightly longer than apical, both nearly straight, phallus apex widening into plate-like structure.

###### Distribution.

Panama.

###### Etymology.

The species name *escobillla* (brush) derives from Spanish, referring to the brush-like apex of the dorsolateral hook in lateral view. The name is a noun in the nominative singular standing in apposition.

##### ﻿ Metrichia leahae sp. nov.

Taxon classificationAnimaliaTrichopteraHydroptilidae

5ACF474F-0728-5C6D-8E99-C8AC0CF79D9F

https://zoobank.org/D132D72E-14A5-4E96-9385-1D2CD450B371

Fig. 13

###### Type locality.

**Panama: Veraguas Province**: Cuenca 097; Santa Fe District; Santa Fe NP; Quebrada sin nombre; PSPSCB-PNSF-C-097-2017-006; 8.55038°N, 81.16486°W; 515 m a.s.l.

###### Type specimen.

***Holotype*: male, Panama: Veraguas Province**: Cuenca 097; Santa Fe District; Santa Fe NP; Quebrada sin nombre; PSPSCB-PNSF-C-097-2017-006; 8.55038°N, 81.16486°W; 515 m a.s.l.; UV light trap; A. Cornejo,T. Ríos, E. Álvarez, C. Nieto, leg.; 20.iv.2017; MIUP-011-T-2023 (in alcohol). ***Paratypes***: Same data as for the holotype, 10 males; MIUP-5, MUPADI-5 (in alcohol).

###### Other material examined.

**Panama: Veraguas Province** • 1 male, Cuenca 097; Santa Fe District; Santa Fe NP; Río Calovébora; PSPSCB-NPSF-C-097-2017-005; 8.54318°N, 81.16398°W; 536 m a.s.l.; Malaise trap; T. Ríos, E. Álvarez, C. Nieto, leg.; 19–23.iv.2017 • ibid., 1 male, UV light trap; 19–21.iv.2017 • ibid., 1 male, Quebrada sin nombre; PSPSCB-NPSF-C-097-2017-011; 8.55343°N, 81.17675°W; 395 m a.s.l.; UV light trap; A. Cornejo, T. Ríos, E. Álvarez, C. Nieto, leg.; 20.iv.2017; • ibid., 1 male, Cuenca 132; Santa Fe District; Santa Fe NP; Quebrada Primer Brazo Mulabá; PSPSCB-PNSF-C132-2017-007; 8.52577°N, 81.13045°W; 623 m a.s.l.; Malaise trap; A. Cornejo, T. Ríos, E. Álvarez, C. Nieto, leg.; 19–23.iv.2017 • 1 male, Quebrada Segundo Brazo Mulabá; Santa Fe NP; PSPSCB-PNSF-C132-2017-010; 8.52906°N, 81.13943°W; 662 m a.s.l.; UV light trap; T. Ríos, E. Álvarez, C. Nieto, leg.; 19.iv.2017 • ibid., 2 males, Quebrada Tercer Brazo Mulabá; Santa Fe NP; PSPSCB-PNSF-C132-2017-014; antes de caseta MiAmbiente; 8.53143°N, 81.14975°W; 746 m a.s.l.; UV light trap; T. Ríos, E. Álvarez, C. Nieto, leg.; 21.iv.2017 • ibid., 64 males, Cuenca 097; Río Piedra de Moler; PSPSCB-PNSF-C097-2017-012; 8.56553°N, 81.18817°W; 340 m a.s.l.; UV light trap; A. Cornejo, T. Ríos, E. Álvarez, C. Nieto, leg.; 20.iv.2017.

###### Diagnosis.

This species has a number of features in common with other *Metrichia*, but the combination of character states renders it unique. The triangular inferior appendage with a rounded apex, in lateral view, resembles those of *M.bracui* Santos,Takiya & Nessimian and several members of the *Metrichianeotropicalis* group (Flint 1983), such as *M.patagonica* Flint. However, the phallus of *M.leahae* sp. nov. has a pair of apical spines, absent in those species with a triangular inferior appendage, but similar to those seen in *M.longitudinis* Bueno-Soria, *M.gombosa* Oláh & Johanson, and *M.kocka* Oláh & Johanson all of which have a differently shaped inferior appendage.

###### Description.

**Male.** Total length 1.8–2.2 mm, antenna broken, forewings with white band at midlength, body brown in alcohol. Abdominal segment V with pair of setose glands on dorsum. Segment VI with pair of medial sacs on dorsum, lateral finger-like glands which telescope. ***Genitalia*.** Segment VII with pair of setose glands on dorsum, laterally wide dorsally tapering ventrad; in ventral view narrow, with broad incision on posterior margin. Segment IX in lateral view triangular, narrow, and truncate posteriorly, tapering anteriorly into segment VI. Preanal appendage (cercus) short and rounded distally in lateral view; in dorsal view oval in shape. Dorsolateral hook in lateral view thin, slightly downturned apically; in dorsal view narrow over length, preapical spine on outer margin. Segment X thin and shelf-like in lateral view; in dorsal view rectanguloid. Inferior appendage triangular in lateral view, tapering distally to rounded apex; in ventral and dorsal views oval in shape with lateral margins lightly sclerotized. Phallus in dorsal view thin and elongate, pair of apical spines, lowermost spine curving outward, upper spine short and straight; in lateral view, subapical spine curving upward and apical spine, separated and curving downward.

**Figure 13.**
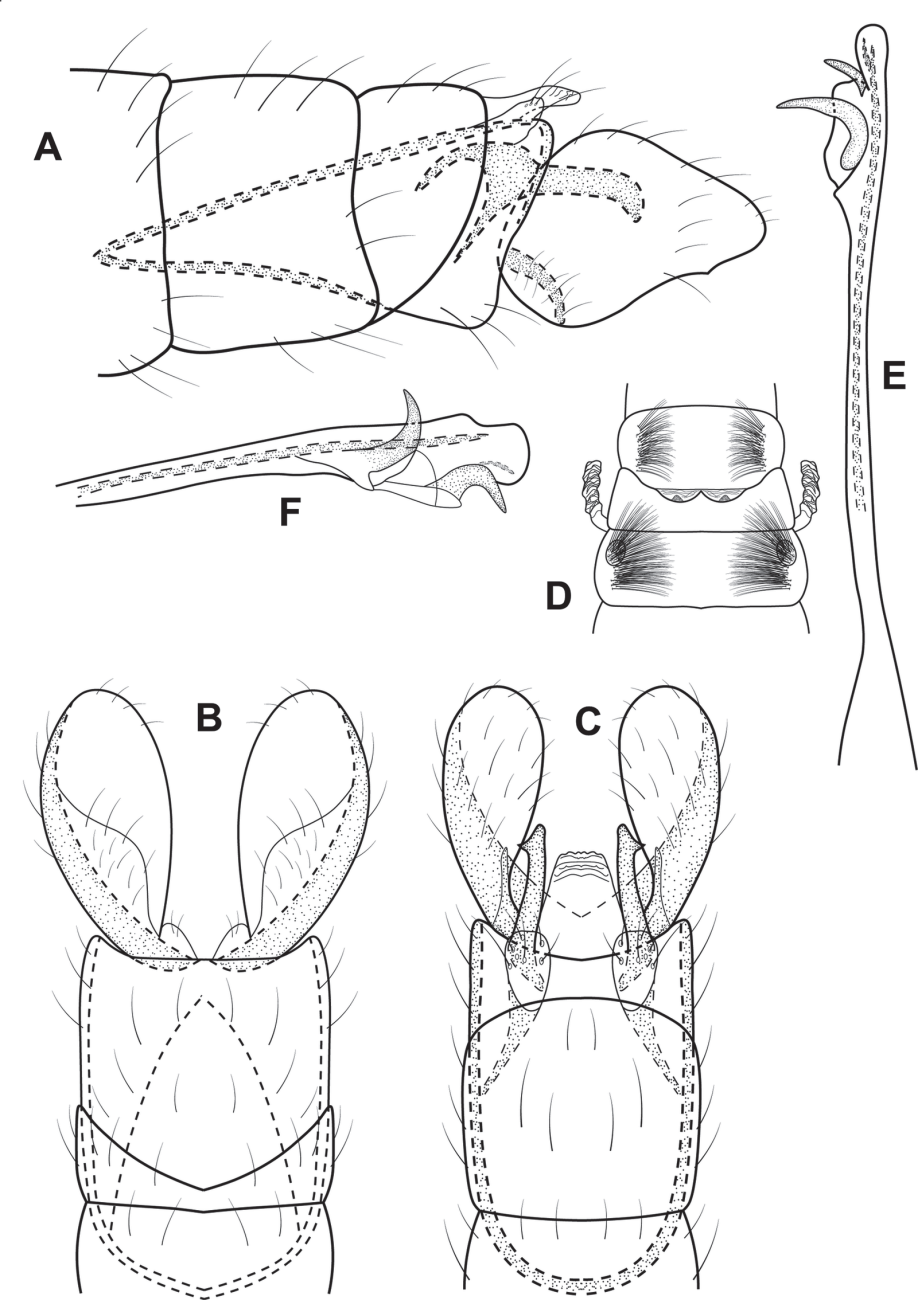
*Metrichialeahae* sp. nov., male holotype, genitalia **A** left lateral **B** ventral **C** dorsal **D** abdominal tergites V–VII, dorsal **E** phallus, dorsal **F** phallus apex, left lateral.

###### Distribution.

Panama.

###### Etymology.

This species is named for Leah Keth, who completed many of the illustrations in this paper and in many others in previous manuscripts for the authors.

##### ﻿ Metrichia tatianae sp. nov.

Taxon classificationAnimaliaTrichopteraHydroptilidae

56B7A3B4-BF91-553E-AD2C-DAE42EBFD077

https://zoobank.org/289C5913-D8AF-4602-8B02-1691413574B3

Fig. 14

###### Type locality.

**Panama: Panama Oeste Province**: Cuenca 138; Chame District; Altos de Campana NP; Río Sajalices; PSPSCB-PNAC-C115-2018-030; 8.67625°N, 79.89748°W; 194 m a.s.l.

###### Type specimen.

***Holotype*: male, Panama: Panama Oeste Province**: Cuenca 138; Chame District; Altos de Campana NP; Río Sajalices; PSPSCB-PNAC-C115-2018-030; 8.67625°N, 79.89748°W; 194 m a.s.l.; UV light trap; E. Pérez, C. Nieto, M. Molinar, T. Ríos, leg.; 29.v.2018; MIUP-012-T-2023 (in alcohol).

###### Diagnosis.

This species shares a number of character states with *M.haranga* Oláh & Johanson from Peru, both of which have segment X elongate and dorsolateral hooks, as well as a pair of apical phallic spines. However, in *M.haranga* these phallic spines are subapical in position, compared to apical in *M.tatianae* sp. nov., and the inferior appendage in *M.tatianae* sp. nov. is not triangular as seen in *M.haranga*, but rather ovoid with the posterior margin incised and much more spinose.

###### Description.

**Male.** Total length 2.1 mm, 20 antennal segments, wings and body brown in alcohol, abdominal terga without modifications. ***Genitalia*.** Abdominal segment VII annular with short ventromesal process. Segment VIII triangular, tapering ventrally; in ventral view deeply incised mesally; in dorsal view quadrate. Segment IX in lateral view triangular, truncate posteriorly, tapering anteriorly into segment VII. Preanal appendage (cercus) oval in lateral and dorsal views. Dorsolateral hook in lateral view very long, thin, tapering to acute apex; in dorsal view wide basally, tapering to acute apices. Segment X very long in lateral view, wide basally and setose, then tapering to acute apex; in dorsal view triangular, apex divided into two tapering processes. Inferior appendage in lateral view somewhat quadrate, narrow at base then widening dorsally, tapering distally to apex which is deeply incised on posterior margin; in dorsal view ovoid, numerous peg-like setae on the mesal surface; in ventral view inner margins diverging, outer margins curved, posterior margins with pointed process. Phallus in dorsal view thin and elongate, pair of separated apical spines mesally; in lateral view, subapical spine shorter than apical spine, both curving upward, phallus apex with small sclerotized spike.

**Figure 14.**
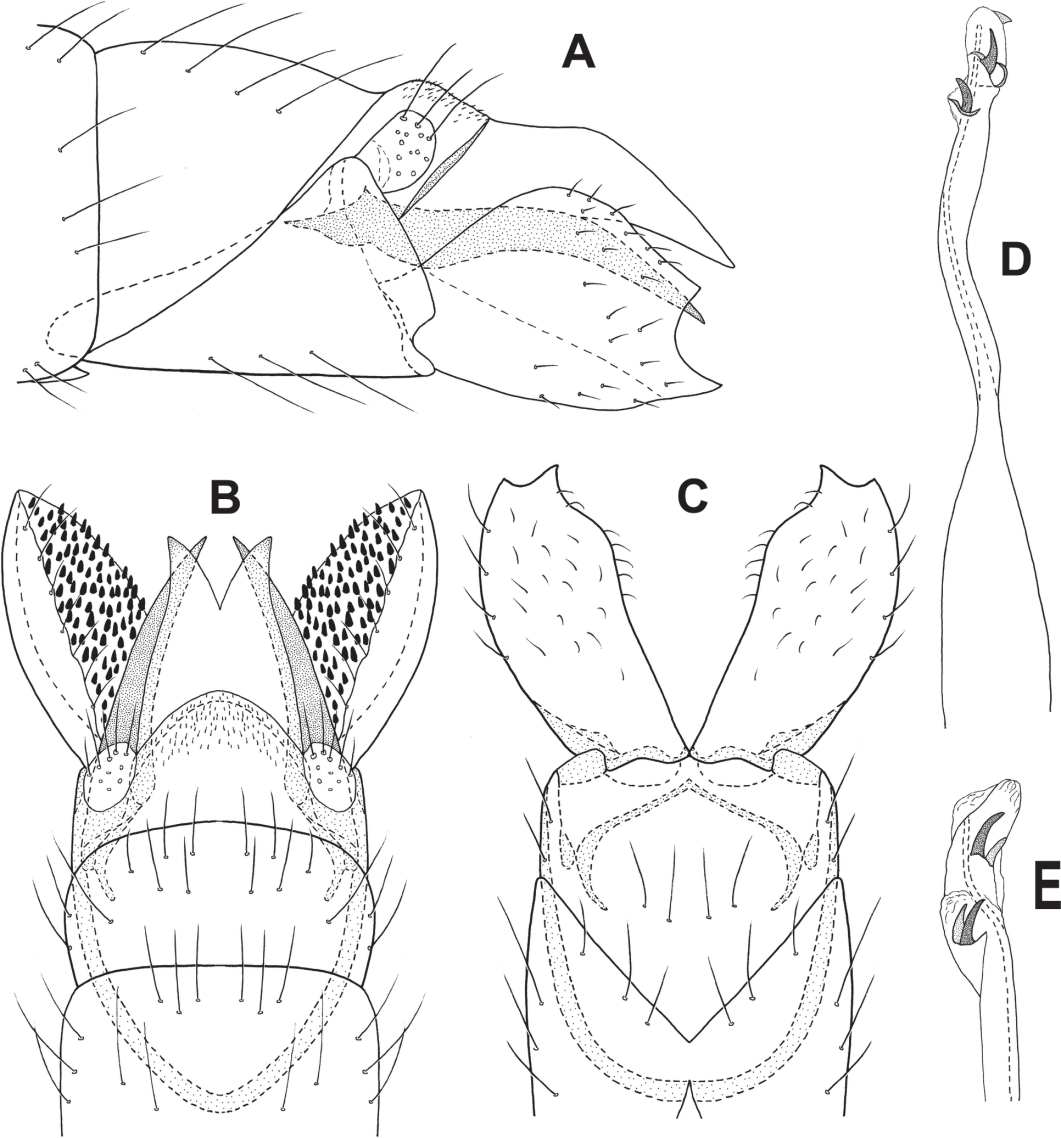
*Metrichiatatianae* sp. nov., male holotype, genitalia **A** left lateral **B** dorsal **C** ventral **D** phallus, dorsal **E** phallus apex, left lateral.

###### Distribution.

Panama.

###### Etymology.

This species is named for Tatiana I. Arefina-Armitage, who completed many of the illustrations in this paper and also did much of the editorial work. We also recognize her lifetime of contributions to Trichopterology.

#### Genus *Ochrotrichia* Ross

The genus *Ochrotrichia* (Hydroptilinae: Ochrotrichiini) is represented by at least 221 extant species (Thomson 2023) endemic to the New World and distributed in North, Central, and South America and the West Indies (Cavalcante et al. 2018; Thomson and Armitage 2018; Harris and Armitage 2019). Previously, 37 species were recorded from Panama (Flint 1970; Armitage et al. 2015, 2016, 2022a; Harris and Armitage 2019; Thomson and Armitage 2021; Harris et al. 2023). Herein we describe and figure two new species.

##### ﻿ Ochrotrichia conejoreja sp. nov.

Taxon classificationAnimaliaTrichopteraHydroptilidae

21EAB8B3-F7F7-5304-AE03-3EE83017A5F3

https://zoobank.org/07DD3DD5-A32C-4084-864D-334E98FC953A

Fig. 15

###### Type locality.

**Panama: Veraguas Province**: Cuenca 097; Santa Fe District; Santa Fe NP; Río Calovébora; PSPSCB-NPSF-C-097-2017-005; 8.54318°N, 81.16398°W; 536 m a.s.l.

###### Type specimen.

***Holotype*: male, Panama: Veraguas Province**: Cuenca 097; Santa Fe District; Santa Fe NP; Río Calovébora; PSPSCB-NPSF-C-097-2017-005; 8.54318°N, 81.16398°W; 536 m a.s.l.; Malaise trap; T. Ríos, E. Álvarez, C. Nieto, leg.; 19-23.iv.2017; MIUP-013-T-2023 (in alcohol).

###### Diagnosis.

This unusual species with a large, flap-like inferior appendage is most similar to *O.unica* Bueno-Soria & Santiago-Fragoso from Columbia and *O.legeza* Oláh & Johanson from Peru, which are similar in the lateral aspect. It differs in the size of the lateral process of the inferior appendage, the structure of tergum X, and the angled phallic apex.

**Figure 15.**
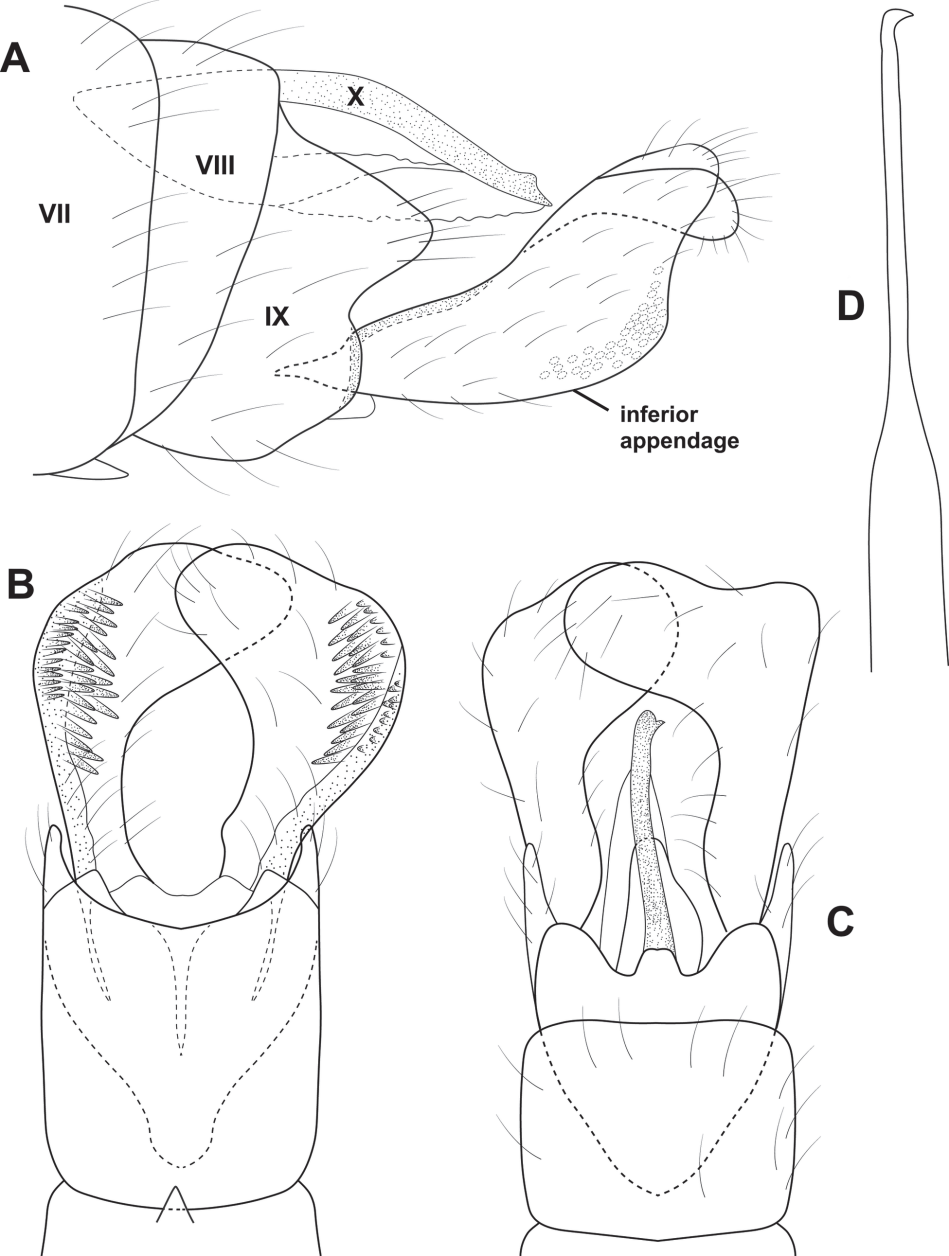
*Ochrotrichiaconejoreja* sp. nov., male holotype, genitalia **A** left lateral **B** ventral **C** dorsal **D** phallus, dorsal.

###### Description.

**Male.** Total length 3.0 mm, antennae long with 38 antennal segments, wings and body brown in alcohol. ***Genitalia*.** Abdominal segment VII annular with short ventromesal process. Segment VIII in lateral view wide dorsally, tapering ventrad on posterior margin; quadrate in dorsal view. Segment IX rectangular in lateral view, incomplete dorsally sinuate posteriorly; in ventral view reduced dorsally, tapering anteriorly; in dorsal view incised on lateral margins. Segment X triangular in lateral view, with sclerotized dorsal plate; in dorsal view triangular, thin, sclerotized, mesal rod with lateral spike apically. Inferior appendage in lateral view narrow at base, widening at midlength and bearing numerous peg-like setae on inner margin, then angled dorsad and tapering distally to rounded apex, large lobe on inner surface subapically; in ventral and dorsal views, narrow basally, greatly widening distally and turning inward, apices overlapping, numerous peg-like setae on lateral margins. Phallus in dorsal view thin over length, but wider basally, apex angled forming short spike.

###### Distribution.

Panama.

###### Etymology.

The species name *conejoreja* (rabbit-eared) derives from Spanish, referring to the inferior appendage, which reminded the authors of a rabbit ear.

##### ﻿ Ochrotrichia paraflagellata sp. nov.

Taxon classificationAnimaliaTrichopteraHydroptilidae

A2036839-27DD-54D1-BDC8-B9A4EEC9B77D

https://zoobank.org/9CBE7DCF-6F40-4158-ACEA-9550BBEDDF35

Fig. 16

###### Type locality.

**Panama: Veraguas Province**: Cuenca 097; Santa Fe District; Santa Fe NP; Quebrada sin nombre; PSPSCB-PNSF-C-097-2017-006; 8.55038°N, 81.16486°W; 515 m a.s.l.

###### Type specimen.

***Holotype*: male, Panama: Veraguas Province**: Cuenca 097; Santa Fe District; Santa Fe NP; Quebrada sin nombre; PSPSCB-PNSF-C-097-2017-006; 8.55038°N, 81.16486°W; 515 m a.s.l.; UV light trap; A. Cornejo,T. Ríos, E. Álvarez, C. Nieto, leg.; 20.iv.2017; MIUP-014-T-2023 (in alcohol).

###### Other material examined.

**Panama**: Veraguas Province • 2 males, Río Calovébora; Santa Fe District; Santa Fe NP; PSPSCB-NPSF-C-097-2017-005; 8.54318°N, 81.16398°W; 536 m a.s.l.; Malaise trap; T. Ríos, E. Álvarez, C. Nieto, leg.; 19–23.iv.2017; MUPADI.

###### Diagnosis.

This species is most similar to *O.flagellata* Flint and *O.birdae* Harris & Armitage, both of which occur in Panama. It differs from these species primarily in the appearance of the phallic apex. In *O.flagellata* the phallus apex has a prominent loop, in *O.birdae* this loop is absent, and in the new species this loop is replaced by two short acute processes. Additionally, the apex of the tenth tergite in *O.paraflagellata* sp. nov. is asymmetrical, but it is symmetrical in the other two species and there is a ventromesal process from abdominal segment VII which is not found in the other two species.

**Figure 16.**
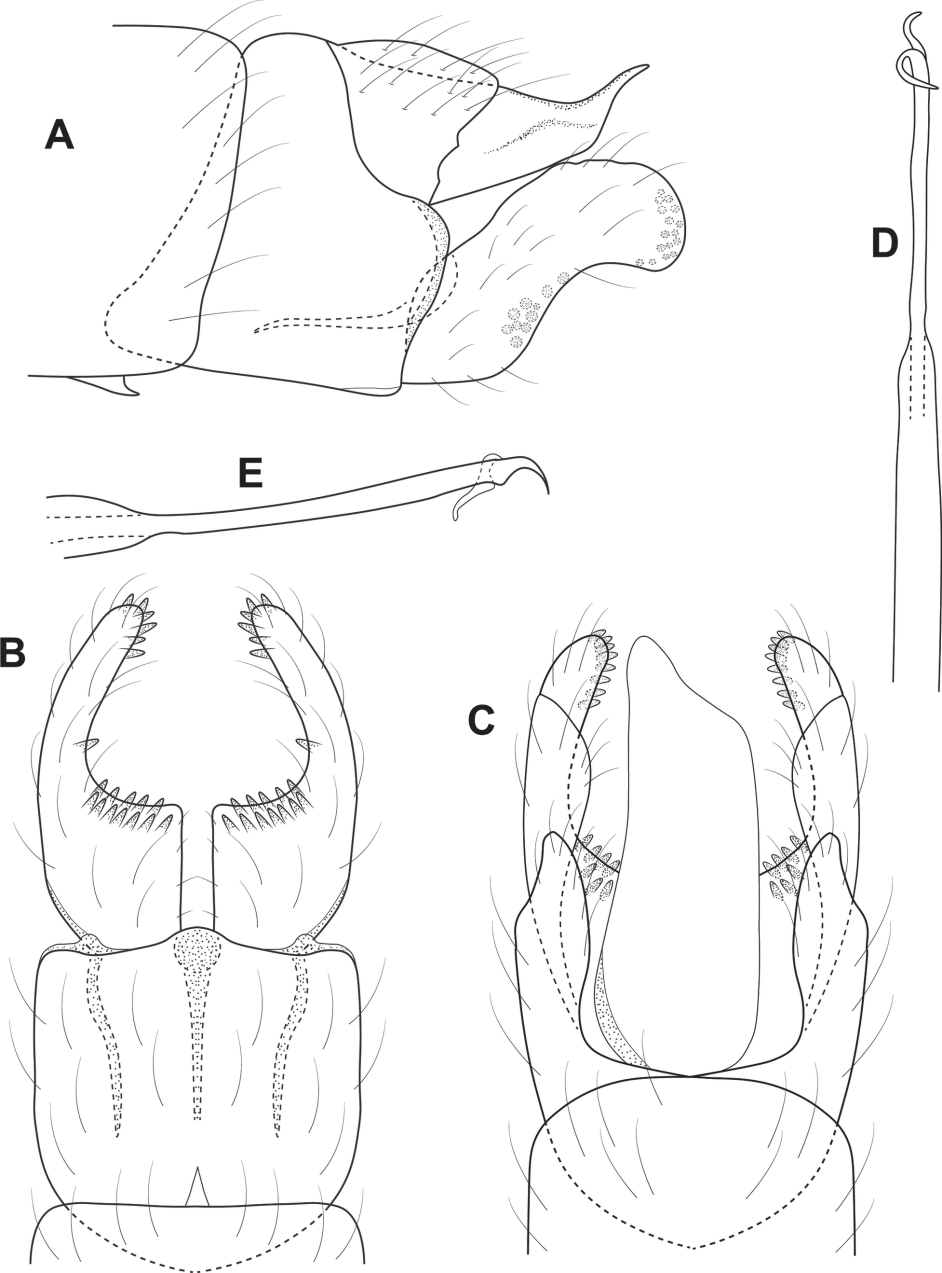
*Ochrotrichiaparaflagellata* sp. nov., male holotype, genitalia **A** left lateral **B** ventral **C** dorsal **D** phallus, dorsal **E** phallus, left lateral.

###### Description.

**Male.** Total length 2.2–2.4 mm, 28 antennal segments, wings and body brown in alcohol. ***Genitalia*.** Abdominal segment VII annular with short ventromesal process. Segment VIII in lateral view truncate posteroventrally, narrowing dorsad, anterior margin tapering ventrad; reduced dorsally and apparently fused with IX, ventrally generally quadrate. Segment IX greatly reduced laterally; in dorsal view deeply incised, producing elongate lateral lobes. Segment X elongate in lateral view, wide basally, narrowing posteriorly, to narrow acute apex; in dorsal view rectangular, apex narrowing on one side to rounded apex. Inferior appendage parallel-sided, curving at midlength to rounded apex, numerous peg-like setae on inner margin; in ventral view, wide basally, curving on inner margin to rounded apex, peg-like setae at base and apex. Phallus in lateral and dorsal view thin over length, but wider basally, apex divided into pair of short acute processes, one projecting forward, the other backwards.

###### Distribution.

Panama.

###### Etymology.

The species name *paraflagellata* (flagellata-like) derives from Spanish, referring its resemblance to *Ochrotrichiaflagellata*.

#### Genus *Oxyethira* Eaton

A member of the Hydroptilinae, *Oxyethira* is cosmopolitan in distribution. Currently, there are about 100 species in the Neotropics, including the Greater and Lesser Antilles (Holzenthal and Calor 2017). Sixteen species are known from Panama, with ten of those added since 2015 (Armitage et al. 2015, 2016; Harris and Armitage 2019). Herein we describe and illustrate one additional species from Panama.

##### ﻿ Oxyethira pehrssonae sp. nov.

Taxon classificationAnimaliaTrichopteraHydroptilidae

8C793D3A-7300-5A68-BFBD-0322B304C523

https://zoobank.org/B9BEB261-6BDB-4286-B680-FBAFCC533BBC

Fig. 17

###### Type locality.

**Panama: Veraguas Province**: Cuenca 132; Santa Fe District; Santa Fe NP; Lago cabaña Alto de Piedra; PSPSCB-PNSF-C132-2017-013; Lago cabaña Alto de Piedra; 8.51423°N, 81.11679°W; 859 m a.s.l.

###### Type specimen.

***Holotype*: male, Panama: Veraguas Province**: Cuenca 132; Santa Fe District; Santa Fe NP; Lago cabaña Alto de Piedra; PSPSCB-PNSF-C132-2017-013; Lago cabaña Alto de Piedra; 8.51423°N, 81.11679°W; 859 m a.s.l.; UV light trap; T. Ríos, E. Álvarez, C. Nieto, leg.; 20.iv.2017; MIUP-015-T-2023 (in alcohol).

###### Diagnosis.

This species is a member of the *Oxyethiraaeola* group of the subgenus Oxytrichia, with closest similarity to *O.apinolada* Holzenthal & Harris. It differs from this species, which occurs in Panama and neighboring Costa Rica, in the ear-like appearance of the subgenital plate in dorsal view, the distinct presence of segment X, and the prominent bilobed process which is absent or indistinct in *O.apinolada*.

**Figure 17.**
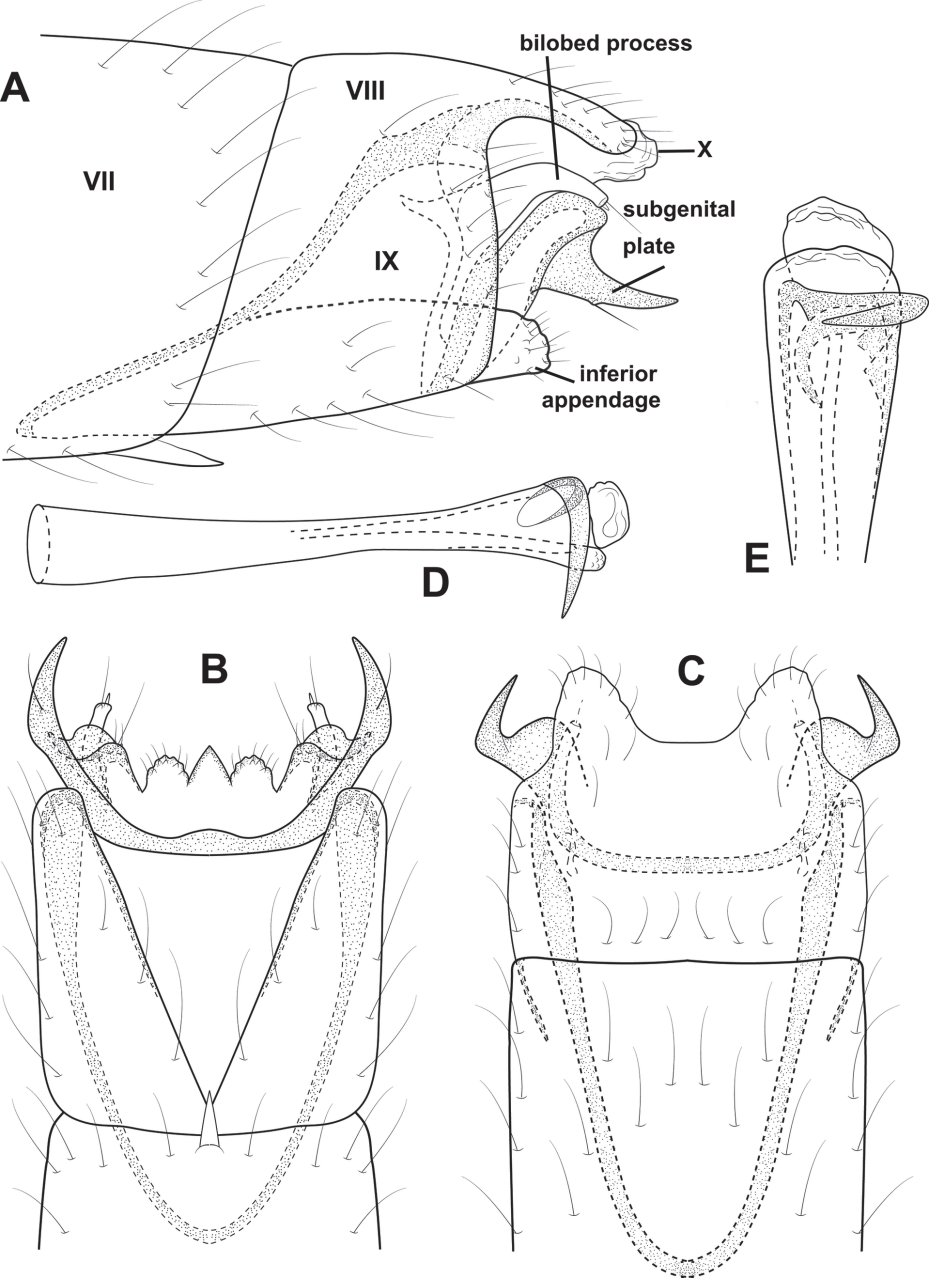
*Oxyethirapehrssonae* sp. nov., male holotype, genitalia **A** left lateral **B** ventral **C** dorsal **D** phallus, left lateral **E** phallus apex, ventral.

###### Description.

**Male.** Total length 2.5 mm, 27 antennal segments, wings and body brown in alcohol. ***Genitalia*.** Abdominal segment VII annular, with short ventromesal process. Segment VIII with prominent, dorsoapical shelf, margin straight ventrad; in dorsal view, broadly incised posteriorly; in ventral view with deep, V-shaped mesal incision. Segment IX largely contained within VIII, tapering anteriorly into segment VII, posteriorly with narrow, shelf-like lobe. Segment X membranous, extending slightly beyond segment VIII in lateral view. Inferior appendage in lateral view narrow, apex rounded; in ventral view with pair of rounded lobes adjacent to triangular mesal lobe. Subgenital plate tapering distally to acute apex; in ventral view narrow, curving outward to acute apices; in dorsal view, falcate, bent sharply at midlength to acute apices. Bilobed process elongate laterally, wide basally tapering posteriorly and bearing an elongate seta; in dorsal and ventral views narrow over length. Phallus wide basally and apically, apex with heavily sclerotized spine nearly encircling shaft.

###### Distribution.

Panama.

###### Etymology.

This species is named for Dr Dale-Elizabeth Pehrsson, former President of the Pennsylvania Western University, which includes Clarion University, in recognition of her leadership and support of scholarship at these institutions.

#### Genus *Zumatrichia* Mosely

A moderately large, Neotropical genus with approximately 54 species (Thomson 2023; Armitage and Harris 2023), *Zumatrichia* (Leucotrichiinae) is distributed from Mexico, through Central America, and into northern South America and the Caribbean Islands. Panama is home to 21 species (Thomson 2023; Armitage and Harris 2023) and serves as the type country for 14 of those taxa. Herein we describe and figure one new species.

##### ﻿ Zumatrichia culebra sp. nov.

Taxon classificationAnimaliaTrichopteraHydroptilidae

2525B813-C8FB-5CAF-8E66-6F749BC3D756

https://zoobank.org/1C3F1035-1DC2-4379-B270-D77D3B9155FE

Fig. 18

###### Type locality.

**Panama: Veraguas Province**: Cuenca 097; Santa Fe District; Santa Fe NP; Quebrada sin nombre; PSPSCB-PNSF-C-097-2017-006; 8.55038°N, 81.16486°W; 515 m a.s.l.

###### Type specimen.

***Holotype*: male, Panama: Veraguas Province**: Cuenca 097; Santa Fe District; Santa Fe NP; Quebrada sin nombre; PSPSCB-PNSF-C-097-2017-006; 8.55038°N, 81.16486°W; 515 m a.s.l.; UV light trap; A. Cornejo,T. Ríos, E. Álvarez, C. Nieto, leg.; 20.iv.2017; MIUP-016-T-2023 (in alcohol). ***Paratypes***: 6 males, same information as for the holotype; MIUP-3, MUPADI-3; in alcohol • ibid., Malaise trap; 23–27.iv.2017; T. Ríos, E. Álvarez, C. Nieto, 1 male, MIUP (in alcohol).

###### Other material examined.

**Panama: Veraguas Province** • Cuenca 097; Santa Fe District; Santa Fe NP; Río Calovébora; PSPSCB-NPSF-C-097-2017-005; 8.54318°N, 81.16398°W; 536 m a.s.l.; UV light trap; T. Ríos, E. Álvarez, C. Nieto, leg.; 19–23.iv.2017; 65 males; ibid., Malaise trap; T. Ríos, E. Álvarez, C. Nieto, leg.; 19–23.iv.2017; 1 male • ibid., Río Calovébora; Santa Fe District; Santa Fe NP; PSPSCB-NPSF-C-097-2017-005; 8.54318°N, 81.16398°W; 536 m a.s.l.; UV light trap; T. Ríos, E. Álvarez, C. Nieto, leg.; 21.iv.2017; 14 males • ibid., Río Piedra de Moler; PSPSCB-PNSF-C097-2017-012; 8.56553°N, 81.18817°W; 340 m a.s.l.; UV light trap; A. Cornejo, T. Ríos, E. Álvarez, C. Nieto, leg.; 21.iv.2017; 5 males • ibid., Quebrada sin nombre; PSPSCB-NPSF-C-097-2017-011; 8.55343°N, 81.17675°W; 395 m a.s.l.; UV light trap; A. Cornejo, T. Ríos, E. Álvarez, C. Nieto, leg.; 20.iv.2017; 13 males (MIUP).

###### Diagnosis.

On the basis of the tripartite inferior appendage, this species is placed in the *Zumatrichiagaltena* group of Flint (1970), showing similarity to *Z.attenuata* Flint, *Z.dereka* Oláh & Flint, and *Z.flinti* Harris & Armitage all of which occur in Panama. It differs from these species in the structure of the inferior appendage and the phallus, which has a distinctive sinuate dorsal rod, not seen in the other species.

**Figure 18.**
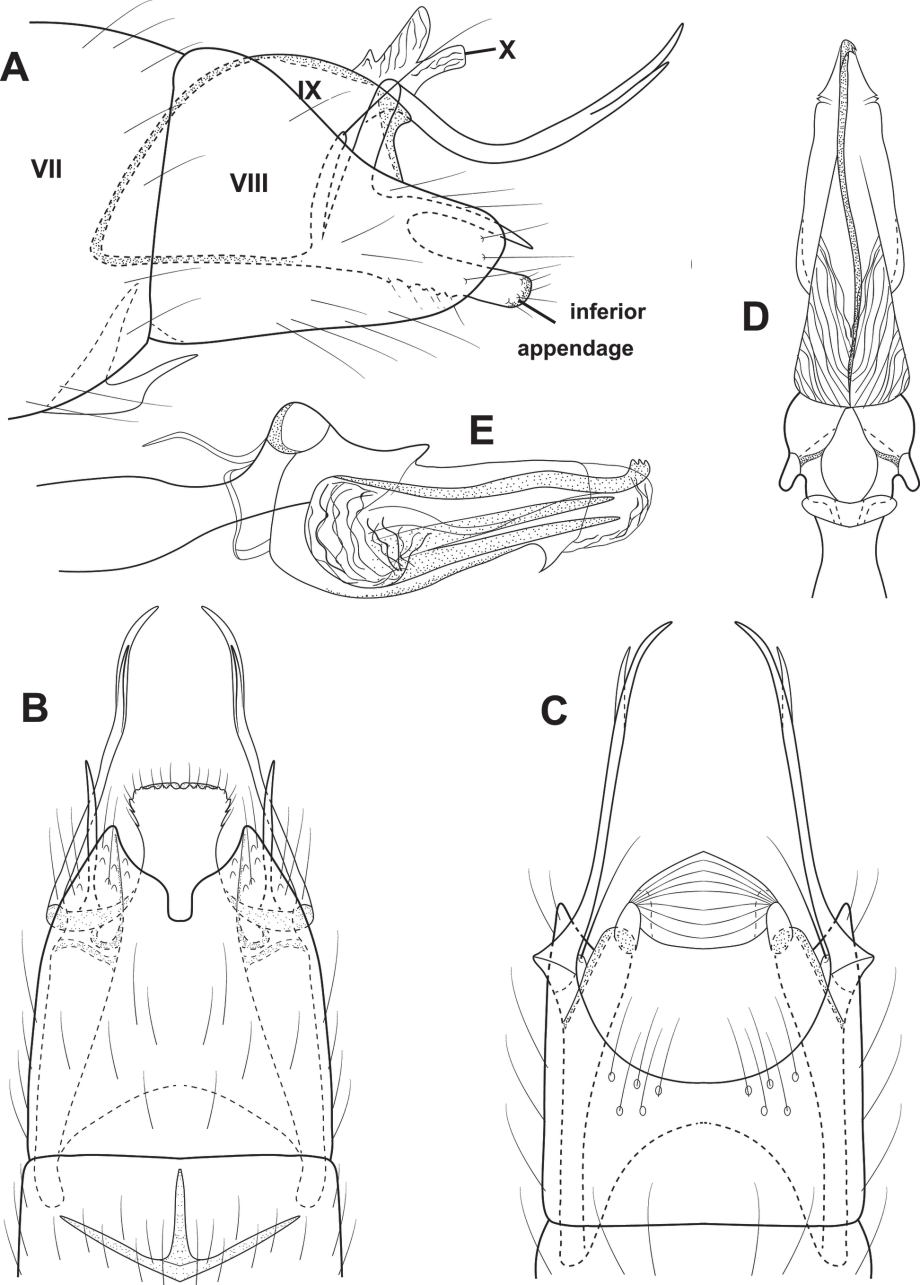
*Zumatrichiaculebra* sp. nov., male holotype, genitalia **A** left lateral **B** ventral **C** dorsal **D** phallus apex, dorsal **E** phallus apex, left lateral.

###### Description.

**Male.** Total length 4.5–5.5 mm, 19 antennal segments with scape enlarged, wings dark brown with while diagonal band near forewing midlength, body brown in alcohol. ***Genitalia*.** Abdominal segment VII annular with ventromesal process. Segment VIII in lateral view truncate posteriorly, tapering anterodorsally; in dorsal view with deep, broad emargination, anteriorly; in ventral view with broad posterior incision, narrowing anteriorly. Segment IX generally quadrate in lateral view, narrowing anteriorly, posterior margin with dorsal lobe, elongate setal-bearing process posteroventrally, mesal incision on posterior margin; dorsally emarginated on posterior and anterior margins. Segment X rectanguloid in lateral aspect, distally bifid and membranous; in dorsal view triangular. Inferior appendage tripartite, dorsalmost process angled dorsally then thin and elongate, with subapical ventral process, medial process thin and acute distally, ventralmost process rectangular, truncate distally; in ventral view this ventralmost process is truncate posteriorly with numerous setae, incised on lateral margins and serrate. Penal sheath with subapical point in lateral view; phallus with medial ring-like structure, posteriorly with pair of elongate ventral spines, dorsally with sinuate process which ends in small incisions; in dorsal view this thin sinuate process extends from the ring-like structure to the phallic apex.

###### Distribution.

Panama.

###### Etymology.

The species name *culebra* (snake) derives from Spanish, referring to the sinuate dorsal rod of the phallus.

## ﻿Discussion

The PSPSCB project, while not as thorough or extensive as originally planned, has produced a large number of new species and new country records of Trichoptera for Panama. The very positive results of these short visits to a variety of sites in a few of Panama’s protected areas provide a strong motive for conducting more extensive surveys at multiple times during the year, employing these same survey methods; this is not the final note because there are a number of macro-Trichoptera which still await description. The results of this project and others in which we have embarked convince us that there are many more new species and first country records awaiting our curiosity and determination.

With the description and publication of the 16 new species here, the total number of Trichoptera species recorded for Panama now stands at 506, an increase of 249 species over the total number (*n* = 257) when we started publishing our results in 2015. Whereas a significant number of macro-Trichoptera have been added to Panama’s fauna, primarily as first country records, the micro-Trichoptera continue to be the group with the most potential for increasing Panama’s species diversity for this order of insects.

## Supplementary Material

XML Treatment for Alisotrichia eisbergae

XML Treatment for Angrisanoia bokota

XML Treatment for Bredinia paraespinosa

XML Treatment for Cerasmatrichia garfioza

XML Treatment for Cerasmatrichia veraguasensis

XML Treatment for Costatrichia calovebora

XML Treatment for Metrichia calovebora

XML Treatment for Metrichia cascada

XML Treatment for Metrichia chiriquiensis

XML Treatment for Metrichia escobilla

XML Treatment for Metrichia leahae

XML Treatment for Metrichia tatianae

XML Treatment for Ochrotrichia conejoreja

XML Treatment for Ochrotrichia paraflagellata

XML Treatment for Oxyethira pehrssonae

XML Treatment for Zumatrichia culebra

## ﻿Additional information

### Author ORCIDs

Steven C. Harris https://orcid.org/0000-0002-6432-7462

Brian J. Armitage https://orcid.org/0000-0003-3182-1533

### Data availability

All of the data that support the findings of this study are available in the main text.

## References

[B1] Aguila Y (1992) Systematic catalogue of the caddisflies of Panama (Trichoptera). In: Quintero D Aiello A (Eds) Insects of Panama and Mesoamerica, Selected Studies.Oxford University Press, Oxford, 532–548.

[B2] Armitage BJ Cornejo A (2015) Orden Trichoptera (Insecta) en Panamá: Listas de especies y su distribución por cuencas y unidades administrativas. Puente Biológico 7: 175–199.

[B3] Armitage BJ Harris SC (2020) The Trichoptera of Panama. XIV. New species of microcaddisflies (Trichoptera: Hydroptilidae) from Omar Torrijos Herrera National Park. Insecta Mundi 0763: 1–19.

[B4] Armitage BJ Harris SC (2023) The Trichoptera of Panama. XXIII. Two new microcaddisfly species (Trichoptera: Hydroptilidae). Annales Zoologici 73(2): 171–177. 10.3161/00034541ANZ2023.73.2.003 PMC10838555 38313333

[B5] Armitage BJ Harris SC Holzenthal RW (2015) The Trichoptera of Panama. I. New Records for Caddisflies (Insecta: Trichoptera) from the Republic of Panama. Insecta Mundi 0435: 1–10.

[B6] Armitage BJ Harris SC Blahnik RJ Thomson RE (2016) The Trichoptera of Panama. IV. New records for caddisflies (Insecta: Trichoptera) from the Republic of Panama. Insecta Mundi 0511: 1–13.

[B7] Armitage BJ Blahnik RJ Harris SC Cornejo A Arefina-Armitage TI (2018) The Trichoptera of Panama. VII. Additional new country records for caddisflies from the Republic of Panama. Insecta Mundi 0614: 1–7.

[B8] Armitage BJ Harris SC Blahnik RJ Thomson RE Ríos González TA Aguirre Y (2020) The Trichoptera of Panama. XIII. Further new country records for caddisflies (Insecta: Trichoptera) from the Republic of Panama. Insecta Mundi 0744: 1–8.

[B9] Armitage BJ Harris SC Ríos TA Aguirre Y Arefina-Armitage TI (2021) The Trichoptera of Panama. XVI. Evaluation of Trichoptera (Insecta) from Omar Torrijos Herrera General Division National Park, Aquatic Insects 43(1): 85–98. 10.1080/01650424.2021.1942496

[B10] Armitage BJ Harris SC Ríos González TA Aguirre EYP Arefina-Armitage TI (2022a) The Trichoptera of Panama. XVII. One new genus record and twelve first species records of microcaddisflies (Trichoptera, Hydroptilidae) from the Republic of Panama. Check List 18(1): 233–239. 10.15560/18.1.233

[B11] Armitage BJ Ríos TA Aguirre EYP Blahnik RJ (2022b) The Trichoptera of Panama. XVIII. Twelve first country records of macrocaddisflies (Insecta: Trichoptera) from the Republic of Panama. Zootaxa 5168(5): 578–588. 10.11646/zootaxa.5168.5.6 36101262

[B12] Blahnik RJ Holzenthal RW (2004) Collection and curation of Trichoptera, with an emphasis on pinned material. Nectopsyche. Neotropical Trichoptera Newsletter 1: 8–20.

[B13] Bueno-Soria J Holzenthal RW (2003) New species and records of the microcaddisfly genus *Metrichia* Ross from Costa Rica (Trichoptera: Hydroptilidae). Studies on Neotropical Fauna and Environment 38(3): 173–197. 10.1076/snfe.38.3.173.28164

[B14] Bueno-Soria J Santiago-Fragoso S (2002) Description of five new species of the genus *Metrichia* Ross (Trichoptera: Hydroptilidae) from Panama. Transactions of the American Entomological Society 128: 245–254.

[B15] Calor AR Mariano R (2012) UV light pan traps for collecting aquatic insects. EntomoBrasilis 5(2): 164–166. 10.12741/ebrasilis.v5i2.187

[B16] Cavalcante BMS Dumas LL Nessimian JL (2018) New species and new geographic record of *Ochrotrichia* Mosely 1934 (Trichoptera: Hydroptilidae) from Río de Janeiro State, Brazil. Zootaxa 4462(2): 229–236. 10.11646/zootaxa.4462.2.4 30314043

[B17] Cornejo A López-López E Ruiz-Picos RA Sedeño-Díaz JE Armitage BJ Arefina TI Nieto C Tuñón A Molinar M Ábrego T Pérez E Tuñón AR Magué J Rodríguez A Pineda J Cubilla J Avila Quintero IM (2017) Diagnóstico de la condición ambiental de los afluentes superficiales de Panamá. Ministerio de Ambiente, 326 pp.

[B18] Flint Jr OS (1970) Studies of Neotropical caddisflies. X: *Leucotrichia* and related genera from North and Central America (Trichoptera: Hydroptilidae). Smithsonian Contributions to Zoology 60(60): 1–64. 10.5479/si.00810282.60

[B19] Flint Jr OS (1972) Studies of Neotropical caddisflies. XIII: the genus *Ochrotrichia* for Mexico and Central America (Trichoptera: Hydroptilidae). Smithsonian Contributions to Zoology 118(118): 1–28. 10.5479/si.00810282.118

[B20] Flint Jr OS (1983) Studies of Neotropical caddisflies. XXXIII: New species from Austral South America (Trichoptera). Smithsonian Contributions to Zoology 377(377): 1–100. 10.5479/si.00810282.377

[B21] Flint Jr OS Harris SC Botosaneanu L (1994) Studies of Neotropical caddisflies. L: The description of *Cerasmatrichia*, new genus, a relative of *Alisotrichia*, with descriptions of new and old species and the larva (Trichoptera: Hydroptilidae). Proceedings of the Biological Society of Washington 107: 360–382.

[B22] Harris SC Armitage BJ (2015) The Trichoptera of Panama. II. Ten new species of microcaddisflies (Trichoptera: Hydroptilidae). Insecta Mundi 0437: 1–17. 10.1080/01650424.2023.2205397

[B23] Harris SC Armitage BJ (2019) The Trichoptera of Panama. X. The Quebrada Rambala drainage, with description of 19 new species of microcaddisflies (Trichoptera: Hydroptilidae). Insecta Mundi 0707: 1–54.

[B24] Harris SC Ríos Gonzalez TA Aguirre EYP (2023) Trichoptera of Panama. XX. Six new microcaddisflies (Trichoptera: Hydroptilidae) and two new country records from Panama. Aquatic Insects 1–23. 10.1080/01650424.2023.2205397

[B25] Holzenthal RW Calor AR (2017) Catalog of the Neotropical Trichoptera (Caddisﬂies). ZooKeys 654: 1–566. 10.3897/zookeys.654.9516 PMC5345355 28331396

[B26] Marshall JE (1979) A review of the genera of the Hydroptilidae (Trichoptera). Bulletin of the British Museum (Natural History). Entomology 39(3): 135–239.

[B27] Oláh J Flint Jr OS (2012) Description of new species in the Leucotrichiini tribe (Trichoptera: Hydroptilidae). Annales Historico-Naturales Musei Nationalis Hungarici 104: 1–83.

[B28] Santos APM Takiya DM Nessimian JL (2016) Integrative taxonomy of *Metrichia* Ross (Trichoptera: Hydroptilidae: Ochrotrichiinae) microcaddisflies from Brazil: descriptions of twenty new species. PeerJ 4: 1–54. 10.7717/peerj.2009 PMC4860326 27169001

[B29] Thomson RE (2023) Catalog of the Hydroptilidae (Insecta, Trichoptera). ZooKeys 1140: 1–499. 10.3897/zookeys.1140.85712 36760708 PMC9871792

[B30] Thomson RE Armitage BJ (2018) The Trichoptera of Panama. VI. Seven new species of microcaddisflies (Insecta: Trichoptera: Hydroptilidae) from Mount Totumas Cloud Forest and Biological Reserve. Insecta Mundi 0613: 1–15.

[B31] Thomson RE Armitage BJ (2021) The Trichoptera of Panama. XV. Six new species and four new country records of microcaddisflies (Insecta: Trichoptera: Hydroptilidae) from Mount Totumas Cloud Forest and Biological Reserve. Revista Mexicana de Biodiversidad 92(0): e923631. 10.22201/ib.20078706e.2021.92.3631

[B32] Thomson RE Armitage BJ Harris SC (2022) The Trichoptera of Panama. XIX. Additions to and a review of the genus *Leucotrichia* (Trichoptera: Hydroptilidae) in Panama. ZooKeys 1111: 425–466. 10.3897/zookeys.1111.77371 36760845 PMC9848940

